# Copper(I)-Photocatalyzed Addition of Trichloromethanesulfenyl Chloride to Olefinic Compounds

**DOI:** 10.3390/molecules30030661

**Published:** 2025-02-02

**Authors:** Nejc Petek, Tilen Zorko, Martin Škrinjar, Uroš Grošelj, Jurij Svete, Drago Kočar, Bogdan Štefane

**Affiliations:** Faculty of Chemistry and Chemical Technology, University of Ljubljana, Večna pot 113, 1000 Ljubljana, Slovenia

**Keywords:** copper photocatalysis, alkenes, sulfenylation, radical chain reaction

## Abstract

Atom transfer radical addition (ATRA) reactions are essential transformations in organic synthetic chemistry that enable the atom-economic difunctionalization of abundant olefin feedstocks. In this way, a rich chemical space can be opened up by well-planned combinations of simple starting materials. To build an efficient photocatalytic transformation, the reactivity of trichloromethanesulfenyl chloride toward alkenes and alkynes was investigated under photocatalytic Cu(I) reaction conditions. In this study, we found that trichloromethanesulfenyl chloride can be added to a series of olefins (such as styrenes and electron-rich and -poor olefins) in the presence of 1 mol% [Cu(dmp)_2_]BF_4_ photocatalyst and blue LED irradiation, producing α-chloro trichloromethylthioethers in good yields. Experimental and theoretical (DFT) mechanistic studies are consistent with the proposed radical chain mechanism of transformation. This study may serve as a valuable reference for the development of new coupling reactions that are economical and highly efficient processes.

## 1. Introduction

Sulfur-containing organic molecules are widely used in various areas of chemistry [1,2,3,4,5,6]. Therefore, the development of new catalytic methods using low-cost, low-loading catalysts is crucial for sustainable chemistry. However, the use of visible light photocatalytic conversions in organic synthesis has become an attractive alternative for the direct vicinal difunctionalization of olefinic compounds, allowing the incorporation of sulfur functionality in one step. Currently, the most commonly used photocatalysts are transition metal complexes based on ruthenium(II) and iridium(III), which exhibit the desired properties for photocatalysts, such as photostability, a long excited-state lifetime, strong absorption in the visible region, and suitable redox potentials [7,8,9,10]. However, disadvantages of this class of photocatalysts include relatively high costs, potential toxicity, and scarcity of the corresponding metal salts [11]. In this context, copper complexes, among others, are becoming increasingly important for visible light-mediated conversion [12]. Since a variety of functionalities can be coordinated to the Cu(II) intermediate species formed during the photoinduced SET process with photoexcited Cu(I)*, the subsequent step of ligand transfer to the radical intermediate in various ATRA reactions is favorable. Given the wide availability of sulfonyl chlorides, several methods for efficient sulfonations of alkenes and alkynes with Cu(I) photocatalysts have been developed. Most methods use α-haloalkyl [13,14,15,16], alkyl, or arylsulfonyl chlorides [17,18,19] as the source of sulfonyl radicals, although there are also some examples which use sulfamoyl radicals in photocatalyzed reactions [20,21,22,23,24].

Nevertheless, electrophilic additions of halogens and related X–Y reagents to alkenes have been a standard procedure since the beginnings of modern organic chemistry. Among these transformations, sulfenyl halides have also been investigated [25,26]. In early studies, halogenated analogs such as CF_3_SCl, CCl_3_SCl, and CF_n_Cl_3-n_SCl proved to be difficult in polar additions with alkenes [27]. Following this early work, Zhang found that the addition of CF_n_Cl_3-n_SCl reagents to alkenes could be efficiently promoted by a catalytic amount of CF_3_CO_2_H, producing the corresponding adducts in good yields. Recently, the visible photoinduced Cu(I)-catalyzed three-component coupling of alkyl halides, olefins, and trifluoromethyltiolate was described, leading to the formation of the corresponding thioethers in good yields [28].

We were interested in further exploring the possibilities and utility of photoinduced, copper-catalyzed coupling reactions with olefins through the formation of two new bonds. Based on the above-mentioned literature and our own experience with the ATRA reaction of sulfonyl and sulfamoyl chlorides, we envisioned that trichloromethanesulfenyl chloride might be a suitable reagent for copper(I)-photoinduced ATRA reactions with alkenes. In this study, we found a series of alkenes that can be chlorotrichloromethylsulfenylated under mild Cu(I)-photoinduced reaction conditions.

## 2. Results and Discussion

We initiated our optimization study by investigating the reaction of 4-chlorostyrene **1a** with trichloromethanesulfenyl chloride as the model substrate (Table 1). After optimization, we found that a complete conversion of **1a** to the adduct **2a** was achieved using 1.5 equiv. of trichloromethanesulfenyl chloride in the presence of 1 mol% of [Cu(dmp)_2_]BF_4_ under 450 nm LED irradiation in anhydrous DCM under Ar atmosphere for 2 h (entry 1). The yield of **2a** depended significantly on the solvent used (Table 1, entries 14–17), with CH_2_Cl_2_ proving to be the most suitable for the conversion. Replacing [Cu(dmp)_2_]BF_4_ with other homoleptic [Cu(dap)_2_]BF_4_ or heteroleptic [Cu(dmp)(Xsantphos)]BF_4_ analogs did not significantly alter the transformation (Table 1, entries 2 and 3). In contrast, Ru(II) and acridinium derivatives were unsuitable photocatalysts for the transformation, yielding the corresponding product with low conversion (Table 1, entries 4 and 5). The control experiments suggested that both the photocatalyst and light are required for high conversion. However, wavelengths longer than 450 nm (e.g., 520 nm) were inefficient. It is worth noting that we did not observe a significant reduction in conversion when the photocatalyst loading was only 0.1 mol% (Table 1, entries 9 and 10). The optimal reaction time was found to be 2 h for most of the substrates studied. Furthermore, we found that moisture and the presence of oxygen significantly decreased the reaction yield of product **2a**.

**Scheme 1:**
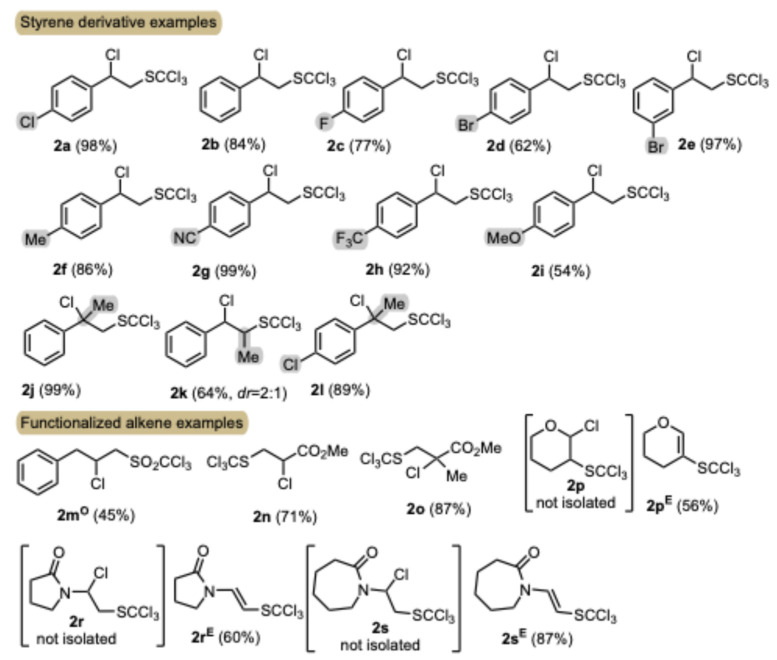
Alkene scope with respect to chlorotrichloromethylsulfonylation.

The established protocol was tested on a series of reactions with a range of alkene substrates bearing different electronic and steric properties (Scheme 1). A variety of functional groups such as halides, nitriles, trifluoromethyl, ethers, and alkyl substituents were tolerated under standard reaction conditions in styrene substrates, with **2** obtained in synthetically useful isolated yields (62–99%). Also, α- or β-alkyl substitution of the alkene moiety in the styrene substrates did not alter the yield of the reaction, and the corresponding products were isolated in good yields (examples **2j**–**2l**). In the case of allylbenzene, product isolation proved to be difficult. Therefore, the corresponding trichloromethanesulfonyl derivative **2m^O^** was isolated in adequate yield after oxidizing the crude product **2m** with H_2_O_2_ to the corresponding sulfone **2m^O^**, which could be easily purified. Fortunately, methyl acrylate and methacrylate, examples of electron-poor alkene substrates, afforded the desired product (**2n**, **2o**) in good 71 and 87% yield, respectively. Vinyl-substituted pyrrolidin-2-one and piperidin-2-one could be used in the addition reaction, as moderate to high yields of products **2r^E^** and **2s^E^** were obtained. In addition, the reactivity of 3,4-dihydro-2*H*-pyran was investigated as an example of an electron-rich olefin, where **2p^E^** was formed in a reasonable yield of 56%. It is worth noting that the corresponding initially formed geminal chloro-heteroatom-substituted adducts **2p**–**2s** were susceptible to an elimination reaction in the applied basic (Et_3_N) reaction workup and consequently the corresponding products of the elimination reaction **2p^E^**–**2s^E^** were isolated instead (Scheme 1).

**Scheme 2:**
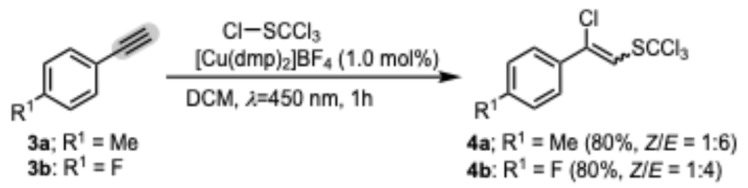
Substituted acetylene examples.

From the examples and mechanistic studies examined (see infra), it is evident that the regioselectivity of the transformation is due to the stability of the corresponding radical intermediate involved in the reaction. It should be emphasized that alkenes with protic functionality present, such as alcohol, thiol, amine, and primary and secondary amide, do not give the desired products due to the rapid reaction under the applied reaction conditions with trichloromethanesulfenyl chloride. Furthermore, in the reaction of trichloromethanesulfenyl chloride with representative alkyne **3** (Scheme 2), the corresponding substituted alkenes were obtained in good yields under optimized reaction conditions. Product **4** was isolated as a mixture of *E*/*Z* isomers, the ratio of which was determined by GC and 1H NMR, with the *E* isomer proving to be the predominant isomer based on the NOESY correlation observed between the olefinic =CH and ortho-phenyl hydrogens in the *Z* isomer.

The structural characterization of products **2** and **4** by ^13^C-NMR spectroscopy revealed a characteristic quaternary –S***C***Cl_3_ ^13^C-NMR resonance occurring in the range 96.9–97.9 ppm, with a long relaxation time. However, the LC-MS experiments showed that most compounds did not ionize under ESI conditions (both the positive and negative mode were tested). Therefore, we cannot report the exact mass of the molecular peak in high resolution for most of product **2**. We found that the compounds ionized in the EI mode readily; therefore, we recorded the mass spectra on a GC-MS instrument and provided experimentally obtained isotopic patterns of the mass spectra at m/z = M for synthesized products **2** and **4**.

**Figure 1:**
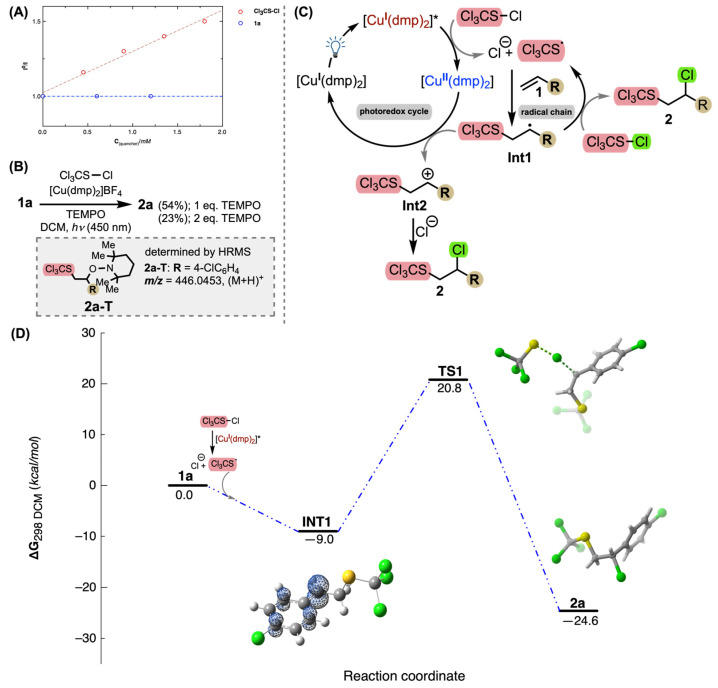
Mechanistic experiments, DFT study, and a plausible reaction mechanism for the chlorotrichloromethylsulfonylation of alkenes: (**A**) Stern–Volmer experiment; (**B**) radical trapping experiment; (**C**) proposed reaction mechanism; and (**D**) DFT calculations of the crucial radical chain reaction steps between the sulfenyl radical intermediate and substrate **1a** and the chlorine abstraction elementary step. The reaction diagram was calculated at the (U)ωB97X-D/Def2-TZVP/SMD(DCM) level of theory. All relative free energies are reported in kcal/mol.

To gain mechanistic insight into the chlorotrichloromethylsulfonylation of olefins by Cu(I) photocatalysis, several control experiments were performed. The Stern–Volmer fluorescence quenching experiment of [Cu(dmp)_2_]BF_4_ was carried out using different concentrations of trichloromethanesulfenyl chloride and **1a**, respectively (Figure 1A). The fluorescence quenches show that [Cu(dmp)_2_]BF_4_ is quenched by sulfenyl chloride and not by **1a**. Thus, considering the reduction potential of [Cu(dmp)_2_]BF_4_^(*)^ (–1.54 V vs. SCE), the one-electron reduction of trichloromethanesulfenyl chloride by the photocatalyst occurs preferentially. A radical trapping experiment was also performed to identify the key intermediates involved in the reaction. Conducting the standard reaction with TEMPO significantly decreased the yield of the reaction, as the TEMPO adduct **2a**-**T** was detected by LC-HRMS analysis of the crude reaction mixture and therefore confirmed the radical intermediate in the reaction (Figure 1B). Moreover, we decided to determine the quantum yield Φ of the reaction to investigate the possible involvement of the radical chain reaction mechanism. The reaction quantum yield for the standard reaction was 68 (greater than 1), indicating that the radical chain process is prominent in this transformation.

In addition to the above experiments, which demonstrated the possibility of an apparently radical chain reaction cycle, the mechanism was investigated computationally using density functional theory (DFT). The reaction of 4-chlorostyrene (**1a**) and the Cl_3_CS^•^ radical was chosen as the model system. DFT calculations were performed at the (U)ωB97X-D/Def2-TZVP/SMD(DCM) level of theory (see Supplementary Materials for details). However, all attempts to identify the corresponding transition state for the first elementary step, the trichloromethylsulfenyl radical addition to **1a**, were not productive. However, the thorough relaxed potential energy surface scan (see Supplementary Materials for details) toward the formation of the S–C bond to form intermediate **INT1** shows that this step is a virtually barrier-free downhill process with an energy of 9.0 kcal/mol. The radical intermediate **INT1** is nearly planar, with most of the spin density localized at the benzylic carbon atom and a partial spin density distribution at the *ortho* and *para* carbon atoms of the phenyl moiety. The abstraction of chlorine from trichloromethylsulfenyl chloride by the benzylic radical intermediate **INT1** involves **TS1** and requires 29.8 kcal/mol of activation energy to generate product **2a** in an exergonic process (Figure 1D).

On the basis of above experimental results, DFT calculations, and the previously reported processes [13,29,30,31], a plausible mechanism of the reaction is shown in Figure 1C. Irradiation of [Cu(dmp)_2_]BF_4_ with visible light produces the excited state [Cu(dmp)_2_]BF_4_* (–1.54 V vs. SCE), which reduces trichloromethanesulfenyl chloride to generate the corresponding radical anion intermediate. The Cl_3_CSCl radical anion undergoes dissociation to form Cl_3_CS^•^ and Cl^–^. The formed thienyl radical is subsequently added to the alkene which gives intermediate **Int1** that enters the radical chain pathway by abstracting chlorine from trichloromethanesulfenyl chloride to give product **2** and regenerating the Cl_3_CS-radical. Although the radical chain mechanism is plausible, the oxidation quenching mechanism could also be involved, in which the radical intermediate **Int1** is oxidized by Cu(II) to generate the carbocation intermediate **Int2**, which is captured by Cl^–^ to generate product **2**.

## 3. Materials and Methods

Photoredox reactions were carried out in a commercial photoreactor (Penn PhD Photoreactor M2, Penn Photon Devices, LLC, Pennsburg, PA, USA). Anhydrous solvents were used. The NMR spectra were recorded in deuterated solvents with Me_4_Si as the internal standard on a Bruker Avance III UltraShield 500 plus instrument (Bruker, Billerica, MA) at 500 MHz for ^1^H and 126 MHz for ^13^C nuclei, respectively, or on a Bruker Ascend neo NMR 600 instrument (Bruker, Billerica, MA, USA) at 600 MHz for ^1^H and 151 MHz for ^13^C nuclei, respectively. Data for ^1^H NMR are reported as chemical shifts (*δ*) in ppm, multiplicity (bs = broad singlet; s = singlet; d = doublet; t = triplet; q = quartet; and m = multiplet), coupling constant, and integration. Data for ^13^C are reported as chemical shift (*δ*) in ppm. Mass spectra were recorded on an Agilent 6224 Accurate Mass TOF LC/MS spectrometer (Agilent Technologies, Santa Clara, CA, USA) and IR spectra on a Bruker FTIR Alpha Platinum spectrophotometer (Bruker, Billerica, MA, USA). Thin-layer chromatography (TLC) was performed on aluminum-backed silica plates (0.2 mm, 60 F254, Sigma-Aldrich, St. Louis, MO, USA). Visualization of TLC (254 nm, Camag, Muttenz, Switzerland) was performed by fluorescence quenching or potassium permanganate stains. Column chromatography (CC) was performed on silica gel (particle size: 35−70 μm, Sigma-Aldrich, St. Louis, MO, USA).

## 4. Conclusions

In the present study, we describe an efficient coupling method of trichloromethanesulfenyl chloride with alkenes and alkynes for the formation of α-chloro trichloromethanethioethers in good yields using an easily accessible Cu(I) photocatalyst. The ability to perform this photoinduced conversion under mild reaction conditions and blue LED irradiation is advantageous compared to related methods. Mechanistic studies, including DFT calculations, support a predominant radical chain reaction triggered by the Cu(I) photocatalyst rather than the alternative ATRA reaction pathway involving oxidative quenching by the photocatalyst.

## Figures and Tables

**Table 1:**
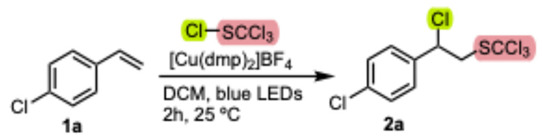
Optimization of the reaction conditions.

Entry	Deviation from Standard Conditions	Yield (%) ^2^
1	none ^1^	99 (98) ^3^
2	[Cu(dmp)(Xsantphos)]BF_4_	97
3	[Cu(dap)_2_]BF_4_	96
4	[Ru(bpy)_3_](PF_6_)_2_	38
5	[Acr^+^-Mes]ClO_4_^–^	10
6	[Cu(dmp)_2_]BF_4_, no light	n.d.
7	[Cu(dmp)_2_]BF_4_, no light, 60 °C	<5
8	520 nm	n.d.
9	[Cu(dmp)_2_]BF_4_, 0.5 mol%	99
10	[Cu(dmp)_2_]BF_4_, 0.1 mol%	87
11	1 min	9
12	0.5 h	82
13	16 h	99
14	MeCN instead of DCM	32
15	toluene instead of DCM	45
16	THF instead of DCM	48
17	1,4-dioxane instead of DCM	50

^1^ Standard reaction conditions: **1a** (0.5 mmol), trichloromethanesulfenyl chloride (0.5 mmol), catalyst ([Cu(dmp)_2_]BF_4_, 1 mol-%), DCM (2 mL), hν (λ = 450 nm), 25 °C, 2 h. ^2^ Yields determined by ^1^H NMR using CH_2_Br_2_ as the internal standard. ^3^ Isolated yield.

## Data Availability

The data presented in this study are available in the main manuscript and in the Supplementary Materials of this manuscript.

## Supplementary Materials

The following supplementary materials can be downloaded at https://www.mdpi.com/article/10.3390/molecules30030661/s1: General procedure for **2** and **4**, ^1^H, ^13^C NMR data, MS spectra of compounds **2** and **4**, reaction quantum yield determination, and details of DFT study.
