# Supplementary material for: Copper(I)-Photocatalyzed Addition of Trichloromethanesulfenyl Chloride to Olefinic Compounds
Source: Molecules. 2025 Feb 2;30(3):661. doi: 10.3390/molecules30030661 (PMC11821038; doi:10.3390/molecules30030661)
Supplement: Supplementary file 1 [file molecules-30-00661-s001.zip › molecules-3422489-supplementary.pdf]

Supporting information

for the article

Copper(I)-photocatalyzed addition of trichloromethanesulfenyl chloride to olefinic compounds

by

Nejc Petek [1], Tilen Zorko [1], Martin Škrinjar [1], Uroš Grošelj [1], Jurij Svete [1], Drago Kočar [1,*] and Bogdan Štefane [1,*]

Faculty of Chemistry and Chemical Technology, University of Ljubljana, Večna pot 113, 1000 Ljubljana, Slovenia

Table of content

General procedure S1

NMR spectra of the new compounds S7

Reaction quantum yield determination S28

Experiment with TEMPO S28

DFT study S29

General procedure for chlorotrichloromethylsulfonylation of alkene substrates.

General Procedure for the Synthesis of Sulfonyl Chlorides 2. A dried 8 mL vial was charged with **1** (0.5 mmol), $[Cu(dmp)_2]BF_4$ (2.9 mg, 0,005 mmol, 1 mol %), CH_2Cl_2 (anhydrous, degassed, 2 mL), and Cl_3CSCl (82 µL, 0.75 mmol). Vial was sealed off with a screw cap with a septum and solution degassed via tree freeze-pump-cycles. The resulting solution was irradiated with LED 450 nm for 2 h at 25 °C. The reaction mixture was quenched with Et_3N (105 µL, 0.75 mol) and solvent evaporated. The pure products **2** were obtained by flash column chromatography (CC) on silica gel 60.

*(2-Chloro-2-(4-chlorophenyl)ethyl)(trichloromethyl)sulfane (**2a**).*

Prepared according to the general procedure from 4-chlorostyrene (**1a**) (60 µL, 0,5 mmol); CC (PE:EtOAc = 1:1); 160 mg (99% yield); colorless oil; νmax/cm^{-1} (ATR) 1596, 1491, 1408, 1091, 1014, 827, 751, 707, 624; ^{1}H NMR (500 MHz, $CDCl_3$) δ 7.37 (*m*, 4H), 5.27 (*t*, *J* = 7.6 Hz, 1H), 3.82 (*dd*, *J* = 13.7, 7.4 Hz, 1H), 3.70 (*dd*, *J* = 13.6, 7.8 Hz, 1H). ^{13}C NMR (500 MHz, $CDCl_3$) δ 137.6, 135.3, 129.4, 128.7, 97.5, 59.0, 45.5; MS (EI) *m/z*: (M^+) [$C_9H_7Cl_5S$]; 321.8 (62%), 323.7 (100%), 325 (64%), 327 (20%).

*(2-Chloro-2-phenyl)ethyl)(trichloromethyl)sulfane (**2b**).*

Prepared according to the general procedure from styrene (**1b**) (58 µL, 0,5 mmol); CC (PE:EtOAc = 1:1); 122 mg (84% yield); colorless oil; *ν*max/cm^{-1} (ATR) 3610, 3164, 3001, 1443, 1375, 1038, 918, 802, 748, 703; ^{1}H NMR (500 MHz, $CDCl_3$) *δ* 7.48–7.33 (*m*, 5H), 5.30 (*t*, *J* = 7.5 Hz, 1H), 3.84 (*dd*, *J* = 13.6, 7.7 Hz, 1H), 3.75 (*dd*, *J* = 13.6, 7.5 Hz, 1H); ^{13}C NMR (500 MHz, $CDCl_3$) *δ* 139.2, 129.4, 129.1, 127.3, 97.6, 60.0, 45.7; MS (EI) *m*/*z*: (M^+) [$C_9H_8Cl_4S$]; 287.8 (78%), 289.8 (100%), 291.8 (48%).

*(2-Chloro-2-(4-fluorophenyl)ethyl)(trichloromethyl)sulfane (**2c**).*

Prepared according to the general procedure from 1-fluoro-4-vinylbenzene (**1c**) (60 µL, 0,5 mmol); CC (PE:EtOAc = 1:1); 119 mg (77% yield); colorless oil; *ν*max/cm^{-1} (ATR) 2978, 1579, 1509, 1408, 1225, 1159, 1097, 836, 762, 709, 624; ^{1}H NMR (500 MHz, $CDCl_3$) *δ* 7.62 (t, *J* = 1.8 Hz, 1H), 7.53 (ddd, *J* = 7.9 Hz, 2.0, 1.0, 1H), 7.39 (dt, *J* = 7.9 Hz, 1.4 Hz 1H), 7.30 (t, *J* = 7.5 Hz, 1H), 5.28 (t, *J* = 7.5 Hz, 1H), 3.84 (dd, *J* = 13.5 Hz, 7.5 Hz, 1H), 3.73 (dd, *J* = 13.5 Hz, 7.5 Hz, 1H); ^{13}C NMR (500 MHz, $CDCl_3$) *δ* 163.0 (d, *J* = 253.9 Hz), 135.1 (d, *J* = 3.6 Hz), 129.2 (d, *J* = 8.5 Hz), 116.1 (d, *J* = 23.0 Hz), 97.5, 59.1, 45.7; MS (EI) *m*/*z*: (M^+) [$C_9H_7Cl_4FS$]; 305.8 (78%), 307.7 (100%), 309.8 (48%).

*(2-(4-Bromophenyl)-2-chloroethyl)(trichloromethyl)sulfane (**2d**).*

Prepared according to the general procedure from 4-bromostyrene (**1d**) (92 µL, 0,5 mmol); CC (PE:EtOAc = 1:1); 113 mg (62% yield); colorless oil; *ν*max/cm^{-1} (ATR) 1590, 1488, 1405, 1246, 1073, 1010, 747, 708. ^{1}H NMR (500 MHz, $CDCl_3$) *δ* 7.56 (*d*, *J* = 8.6 Hz, 2H), 7.34 (*d*, *J* = 8.4 Hz, 2H), 5.29 (*t*, *J* = 7.6 Hz, 1H), 3.84 (*dd*, *J* = 13.7 Hz, 7.4 Hz, 1H), 3.72 (*dd*, *J* = 13.7 Hz, 7.8 Hz, 1H). ^{13}C NMR (500 MHz, $CDCl_3$) *δ* 138.1, 132.3, 129.0, 123.5, 97.5, 59.1, 45.5; MS (EI) *m*/*z*: (M^+) [$C_9H_7BrCl_4S$]; 365.7 (78%), 367.7 (100%), 369.7 (97%), 371.7 (46).

*(2-(3-Bromophenyl)-2-chloroethyl)(trichloromethyl)sulfane (**2e**).*

Prepared according to the general procedure from 3-bromostyrene (**1e**) (91 mg, 0,5 mmol); CC (PE:EtOAc = 1:1); 181 mg (97% yield); colorless oil; *ν*max/cm^{-1} (ATR) 1570, 1474, 1427, 1253, 1194, 1073, 997, 879, 789, 749, 706. ^{1}H NMR (500 MHz, $CDCl_3$) 7.62 (*t*, *J* = 1.9 Hz, 1H), 7.53 (*ddd*, *J* = 7.9 Hz, 2.0 Hz, 1.1 Hz, 1H), 7.39 (*dt*, *J* = 7.8 Hz, 1.4 Hz, 1H), 7.30 (*t*, *J* = 7.9 Hz, 1H), 5.28 (*t*, *J* = 7.5 Hz, 1H), 3.84 (*dd*, *J* = 13.7 Hz, 7.5 Hz, 1H), 3.73 (*dd*, *J* = 13.8 Hz, 7.5 Hz, 1H). ^{13}C NMR (500 MHz, $CDCl_3$) *δ* 141.2, 132.5, 130.6, 130.3, 125.9, 123.0, 97.5, 58.9, 45.4; MS (EI) *m*/*z*: (M^+) [$C_9H_7BrCl_4S$]; 365.7 (78%), 367.7 (100%), 369.7 (97%), 371.7 (46).

*(2-Chloro-2-(p-tolyl)ethyl)(trichloromethyl)sulfane (**2f**).*

Prepared according to the general procedure from 1-methyl-4-vinylbenzene (**1f**) (59 mg, 0,5 mmol); CC (PE:EtOAc = 1:1); 130 mg (86% yield); colorless oil; νmax/cm^{-1} (ATR) 2923, 1579, 1513, 1408, 1245, 1113, 759, 707, 629. ^{1}H NMR (500 MHz, $CDCl_3$) 7.36 (*d*, *J* = 8.2 Hz, 2H), 7.24 (*d*, *J* = 7.8 Hz, 2H), 5.32 (*t*, *J* = 7.6 Hz, 1H), 3.88 (*dd*, *J* = 13.5 Hz, 7.6 Hz, 1H), 3.79 (*dd*, *J* = 13.5 Hz, 7.6 Hz, 1H), 2.41 (*s*, 3H). ^{13}C NMR (500 MHz, $CDCl_3$) δ 139.4, 136.2, 129.8, 127.1, 97.6, 59.9, 45.6, 21.4; MS (EI) *m*/*z*: (M^+) [$C_{10}H_{10}Cl_4S$]; 301.8 (78%), 303.7 (100%), 305.8 (48%).

*4-(1-Chloro-2-((trichloromethyl)thio)ethyl)benzonitrile (**2g**).*

Prepared according to the general procedure from 4-vinylbenzonitrile (**1g**) (60.5 µL, 0,5 mmol); CC (PE:EtOAc = 1:1); 156 mg (99% yield); yelowish oil; νmax/cm^{-1} (ATR) 3621, 2999, 2943, 1442, 1417, 1375, 1038, 918, 845, 801; ^{1}H NMR (500 MHz, $CDCl_3$) δ 7.71 (*d*, *J* = 8.4 Hz, 2H), 7.57 (*d*, *J* = 8.3 Hz, 2H), 5.34 (*t*, *J* = 7.5 Hz, 1H), 3.83 (*dd*, *J* = 13.9, 7.2 Hz, 1H), 3.70 (*dd*, *J* = 13.9, 7.7 Hz, 1H); ^{13}C NMR (500 MHz, $CDCl_3$) δ 143.9, 132.9, 128.2, 118.2, 113.2, 97.3, 58.6, 45.1. MS (EI) *m*/*z*: (M^+) [$C_{10}H_7Cl_4NS$]; 312.7 (78%), 314.8 (100%), 316.8 (48).

*(2-Chloro-2-(4-(trifluoromethyl)phenyl)ethyl)(trichloromethyl)sulfane (**2h**).*

Prepared according to the general procedure from 1-(trifluoromethyl)-4-vinylbenzene (**1h**) (67.0 µL, 0.5 mmol); CC (PE:EtOAc = 1:1); 165 mg (92% yield); yelowish oil; νmax/cm^{-1} (ATR) 2968, 1580, 1519, 1408, 1235, 1162, 1090, 856, 761, 709, 630; ^{1}H NMR (500 MHz, $CDCl_3$) δ 7.67 (*d*, J = 8.2 Hz, 2H), 7.56 (*d*, J = 8.1 Hz, 2H), 5.35 (*t*, J = 7.5 Hz, 1H), 3.84 (*dd*, J = 13.8, 7.4 Hz, 1H), 3.72 (*dd*, J = 13.9, 7.6 Hz, 1H); ^{13}C NMR (500 MHz, $CDCl_3$) δ 142.9, 131.4 (*q*, *J* = 30.0 Hz), 127.8, 126.1 (*q*, *J* = 3.8 Hz), 123.8 (*q*, *J* = 273.0 Hz) 97.5, 58.9, 45.4; MS (EI) *m*/*z*: (M^+) [$C_{10}H_7Cl_4F_3S$]; 355.7 (78%), 357.8 (100%), 359.8 (48).

*(2-Chloro-2-(4-methoxyphenyl)ethyl)(trichloromethyl)sulfane (**2i**).*

Prepared according to the general procedure from 4-methoxystyrene (**1i**) (86 mg, 0,5 mmol); CC (PE:EtOAc = 1:1); 97 mg (54% yield); yellow oil; νmax/cm^{-1} (ATR) 2933, 2836, 1583, 1510, 1461, 1440, 1303, 1246, 1175, 1111, 1031, 960, 831, 799, 707, 623. ^{1}H NMR (500 MHz, $CDCl_3$) δ 7.40–7.37 (*m*, 2H), 6.96–9.93 (*m*, 2H), 5.31 (*t*, *J* = 7.5 Hz, 1H), 3.85 (*s*, 3H), 3.86 (*dd*, J = 13.5, 7.5 Hz, 1H), 3.78 (*dd*, *J* = 13.5, 7.5 Hz, 1H); ^{13}C NMR (500 MHz, $CDCl_3$) δ 160.3, 131.1, 128.6, 114.4, 97.6, 59.8, 55.5, 45.6; MS (EI) *m*/*z*: (M^+) [$C_{10}H_{10}Cl_4OS$]; 317.8 (78%), 319.8 (100%), 321.8 (48).

(2-Kloro-2-fenilpropil)(triklorometil)sulfan (**2j**).

Prepared according to the general procedure from prop-1-en-2-ylbenzene (**1j**) (59.1 mg, 0.5 mmol); CC (PE:EtOAc = 1:1); 151 mg (99% yield); yelowish oil; νmax/cm^{-1} (ATR) 1580, 1494, 1445, 1379, 1037, 755, 692, 651; ^{1}H NMR (500 MHz, $CDCl_3$) δ 7.63–7.62 (m, 2H), 7.45–7.42 (m, 2H), 7.39–7.37 (m, 1H), 3.96 (s, 2H), 2.21 (s, 3H); ^{13}C NMR (500 MHz, $CDCl_3$) δ 142.7, 128.72, 128.68, 126.0, 79.5, 69.8, 52.2, 30.9; MS (EI) *m*/*z*: (M^+) [$C_{10}H_7Cl_4F_3S$]; 301.8 (78%), 303.8 (100%), 305.8 (48%).

*(1-Chloro-1-phenylpropan-2-yl)(trichloromethyl)sulfane (**2k**).*

Prepared according to the general procedure from (*E*)-prop-1-en-1-ylbenzene (**1k**) (59 mg, 0,5 mmol); CC (PE:EtOAc = 1:1); 97 mg (64% yield); yellow oil; νmax/cm^{-1} (ATR) 1493, 1449, 1378, 1052, 798, 761, 696, 636. ^{1}H NMR (500 MHz, $CDCl_3$) δ 7.53–7.49 (m, 2H), 7.45–7.36 (m, 3H), 5.67 (d, *J* = 3.5 Hz, 1H, major izomer), 5.43 (d, *J* = 3.5 Hz, 1H, minor izomer), 3.92–3.84 (m, 1H), 1.68 (*d*, *J* = 7.5 Hz, 3H, minor izomer), 1.52 (d, *J* = 7.0 Hz, 3H, major izomer); ^{13}C NMR (500 MHz, $CDCl_3$) δ 138.4, 136.9, 128.9, 128.7, 128.6, 128.3, 127.9, 127.4, 98.0, 97.6, 67.8, 65.4, 54.6, 54.5, 18.5, 15.5; MS (EI) *m*/*z*: (M^+) [$C_{10}H_7Cl_4F_3S$]; 301.8 (78%), 303.9 (100%), 305.9 (48%).

*(2-Chloro-2-(4-chlorophenyl)propyl)(trichloromethyl)sulfane (**2l**).*

Prepared according to the general procedure from 1-chloro-4-(prop-1-ene-2-yl)benzene (**1l**) (76 mg, 0,5 mmol); CC (PE:EtOAc = 1:1); 152 mg (89% yield); yellow oil; νmax/cm^{-1} (ATR) 2981, 2932, 1579, 1492, 1399, 1378, 1244, 1095, 1038, 1012, 827, 770, 707, 671. ^{1}H NMR (500 MHz, $CDCl_3$) δ 7.55 (*d*, *J* = 8.7 Hz 2H), 7.39 (*d*, *J* = 8.7 Hz, 2H), 3.91 (*s*, 2H) 2.18 (*s*, 3H).^{13}C NMR (500 MHz, $CDCl_3$) δ 141.2, 134.7, 128.8, 127.6, 97.4, 69.0, 52.0, 30.9; MS (EI) *m*/*z*: (M^+) [$C_{10}H_9Cl_5S$]; 335.8 (62%), 337.7 (100%), 339.8 (64%), 341.8 (20).

(2-Chloro-3-((trichloromethyl)sulfonyl)propyl)benzene (**2m^{O}**)

Prepared according to the general procedure from allylbenzene (**1m**) (66.3 µL, 0,5 mmol). The crude product was then reacted in hexafluoro-2-propanol (2 mL) and H_2O_2 (30% w/w, 230 µL, 2 mmol) and purified CC (PE:EtOAc = 20:1); 59 mg (45% yield); yelowish oil; νmax/cm^{-1} (ATR) 1589, 1447, 1407, 1350, 1280, 1214, 1160, 1120, 1078, 953, 790, 761; ^{1}H NMR (500 MHz, $CDCl_3$) δ 7.56–7.09 (*m*, 5H), 4.28 (*ddt*, *J* = 7.5, 6.1, 5.0 Hz, 1H), 3.40 (*dd*, *J* = 14.2, 5.0 Hz, 1H), 3.22 (*dd*, *J* = 14.2, 7.5 Hz, 1H), 3.13 (*m*, 2H); ^{13}C NMR (500 MHz, $CDCl_3$) δ 165.9, 136.2, 129.5, 128.9, 127.5, 60.1, 43.6, 40.8; HRMS (ESI) m/z: $(M+H)^+$, found 236.9202, [$C_{10}H_{11}Cl_4O_2S$]; requires 236.9204.

*Methyl 2-chloro-3-((trichloromethyl)thio)propanoate (**2n**).*

Prepared according to the general procedure from methyl acrylate (**1n**) (43 mg, 0,5 mmol); CC (PE:EtOAc = 1:1); 96 mg (71% yield); yellow oil; *ν*max/cm^{-1} (ATR) 2954, 1745, 1702, 1579, 1437, 1405, 1351, 1275, 1223, 1195, 1166, 1114, 1071, 973, 796, 766, 714; ^{1}H NMR (500 MHz, $CDCl_3$) 4.77 (*dd*, *J* = 8.7 Hz, 6.2 Hz, 1H), 3.85 (*s*, 3H), 3.72 (*dd*, *J* = 14.3 Hz, 8.7 Hz, 1H), 3.59 (*dd*, *J* = 14.3 Hz, 6.2 Hz, 1H); ^{13}C NMR (500 MHz, $CDCl_3$) *δ* 168.4, 97.4, 53.6, 53.2, 40.1; MS (EI) *m*/*z*: (M^+) [$C_5H_6Cl_4O_2S$]; 269.8 (78%), 271.8 (100%), 273.8 (48%).

Methyl 2-chloro-2-methyl-3-((trichloromethyl)thio)propanoate (**2o**)

Prepared according to the general procedure from methyl methacrylate (**1n**) (60.4 μL, 0,5 mmol); CC (PE:EtOAc = 1:1); 125 mg (87% yield); yelowish oil; *ν*max/cm^{-1} (ATR) 2955, 2231, 1737, 1452, 1412, 1380, 1296, 1251, 1206, 1171, 1107, 1052, 984, 799, 765, 715; ^{1}H NMR (500 MHz, $CDCl_3$) *δ* 3.84 (*s*, 3H), 3.79 (*d*, *J* = 12.6 Hz, 1H), 3.72 (*d*, *J* = 12.6 Hz, 1H), 1.94 (*s*, 3H); ^{13}C NMR (500 MHz, $CDCl_3$) *δ* 170.0, 97.4, 65.3, 53.8, 46.9, 27.2; MS (EI) *m*/*z*: (M^+) [$C_6H_8Cl_4O_2S$]; 283.8 (78%), 285.8 (100%), 287.8 (48%).

5-((Trichloromethyl)thio)-3,4-dihydro-2H-pyran (**2p^{E}**)

Prepared according to the general procedure from 3,4-dihydropyran (**1o**) (45.7 μL, 0,5 mmol). The reaction mixture was quenched with Et_3N (105 μL, 0.75 mol) and stirred for 2 h at 25ºC to ensure complete elimination of HCl. CC (PE:EtOAc = 1:1); 66 mg (56% yield); yelowish oil; *ν*max/cm^{-1} (ATR) 3362, 2923, 1640, 1464, 1377, 1071, 860, 721, 652; ^{1}H NMR (500 MHz, $CDCl_3$) *δ* 7.08 (*t*, *J* = 1.5 Hz, 1H), 4.11 (*t*, *J* = 5.5 Hz, 2H), 2.59 (*dt*, *J* = 6.0, 1.5 Hz, 2H), 2.04–1.94 (*m*, 2H); ^{13}C NMR (500 MHz, $CDCl_3$) *δ* 157.4, 106.2, 66.3, 31.1, 27.1, 22.5; HRMS (ESI) m/z: $(M+H)^+$, found 232.9363, [$C_6H_8Cl_3OS$]; requires 232.9361.

(E)-1-(2-((trichloromethyl)thio)vinyl)pyrrolidin-2-one (**2r^{E}**)

Prepared according to the general procedure from 3,4-dihydropyran (**1p**) (53.5 μL, 0,5 mmol). The reaction mixture was quenched with Et_3N (105 μL, 0.75 mol) and stirred for 2 h at 25ºC to ensure complete elimination of HCl. CC (PE:EtOAc = 1:1); 78 mg (60% yield); yelowish oil; *ν*max/cm^{-1} (ATR) 3032, 2925, 1758, 1492, 1410, 1260, 1197, 1075, 1024, 901, 838, 799, 768; ^{1}H NMR (500 MHz, $CDCl_3$) *δ* 7.63 (*d*, *J* = 13.5 Hz, 1H), 5.78 (*d*, *J* = 13.5 Hz, 1H), 3.67 (*t*, *J* = 7.5 Hz, 2H), 2.57 (dd, *J* = 8.5, 7.5 Hz, 2H) 2.27–2.15 (*m*, 2H); ^{13}C NMR (500 MHz, $CDCl_3$) *δ* 173.9, 138.8, 99.5, 98.6, 45.2, 30.9, 17.6; HRMS (ESI) m/z: $(M+H)^+$, found 259.9464, [$C_7H_8Cl_3NOS$]; requires 259.9465.

(E)-1-(2-((Trichloromethyl)thio)vinyl)azepan-2-one (**2s^{E}**)

Prepared according to the general procedure from 1-vinylazepan-2-one (**1r**) (70.0 mg, 0,5 mmol). The reaction mixture was quenched with Et_3N (105 µL, 0.75 mol) and stirred for 2 h at 25ºC to ensure complete elimination of HCl. CC (PE:EtOAc = 1:1); 126 mg (87% yield); white solid; νmax/cm^{-1} (ATR) 2925, 2855, 1714, 1622, 1487, 1436, 1401, 13334, 1286, 1260, 1190, 1083, 979, 735; ^{1}H NMR (500 MHz, $CDCl_3$) δ 7.94 (*d*, *J* = 13.7 Hz, 1H), 5.88 (*d*, *J* = 13.8 Hz, 1H), 3.75–3.62 (*m*, 2H), 2.73–2.64 (*m*, 2H), 1.78 (*dt*, *J* = 11.4, 3.4 Hz, 6H); ^{13}C NMR (500 MHz, $CDCl_3$) δ 174.7, 142.3, 99.9, 97.2, 45.8, 37.1, 29.4, 27.4, 23.5; HRMS (ESI) m/z: $(M+H)^+$, found 287.9773, [$C_9H_{12}Cl_3NOS$]; requires 287.9778.

*(E/Z)-(2-chloro-2-(4-fluorophenyl)vinyl)(trichloromethyl)sulfane (**4a**).*

Prepared according to the general procedure from 1-ethenyl-4-methylbenzene (**3a**) (43 mg, 0,5 mmol); CC (PE:EtOAc = 1:1); 184 mg (80% yield); yellow oil; νmax/cm^{-1} (ATR) 3029, 1579, 1504, 1447, 1404, 1378, 1299, 1212, 1165, 1113, 1020, 914, 771, 715, 658, 638. ^{1}H NMR (500 MHz, $CDCl_3$) 7.32 (*m*, 2H), 7.20 (*m*, 2H), 7.01 (*s*, 0.14H, minor izomer), 6.88 (*s*, 0.86H, major izomer), 2.37 (*s*, 3H); ^{13}C NMR (500 MHz, $CDCl_3$) δ major izomer; 140.6, 132.9, 129.2, 128.6, 126.4, 118.4, 96.9, 21.6; δ minor izomer; 136.7, 136.2, 129.5, 129.0, 128.9, 118,4, 96.9, 21.4; MS (EI) *m/z*: (M^+) [$C_{10}H_8Cl_4S$]; 299.9 (78%), 301.9 (100%), 303.9 (48%).

*(E/Z)-(2-chloro-2-(4-fluorophenyl)vinyl)(trichloromethyl)sulfane (**4b**).*

Prepared according to the general procedure from 1-ethenyl-4-fluorobenzene (**3b**) (43 mg, 0,5 mmol); CC (PE:EtOAc = 1:1); 184 mg (80% yield); yellow oil; νmax/cm^{-1} (ATR) 2977, 1701, 1600, 1578, 1501, 1462, 1405, 1381, 1358, 1299, 1234, 1159, 1098, 1014, 943, 915, 839, 774, 720, 660, 634. ^{1}H NMR (500 MHz, $CDCl_3$) 7.66–7.61 (m, 0.8H, minor izomer) 7.49–7.45 (*m*, 2H), 7.15–7.10(*m*, 2H), 7.03 (*s*, 0,20H, minor izomer), 6.95 (*s*, 0,90H, major izomer); ^{13}C NMR (500 MHz, $CDCl_3$) δ major izomer; 163.5 (*d*, *J* = 252.5 Hz), 135.9, 130.7 (*d*, *J* = 8.5 Hz), 128.3 (*d*, *J* = 10.5 Hz), 119.1, 115.6 (*d*, *J* = 22.0 Hz), 96.7; δ minor izomer; 137.1, 131.7 (*d*, *J* = 4.0 Hz), 131.0 (*d*, *J* = 8.5 Hz), 119.4, 115.7 (*d*, *J* = 22.0 Hz), 96.7; MS (EI) *m/z*: (M^+) [$C_9H_5Cl_4FS$]; 303.8 (78%), 305.9 (100%), 307.9 (48%).

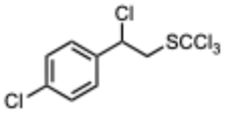
Cl
SCCl3
Cl

2a

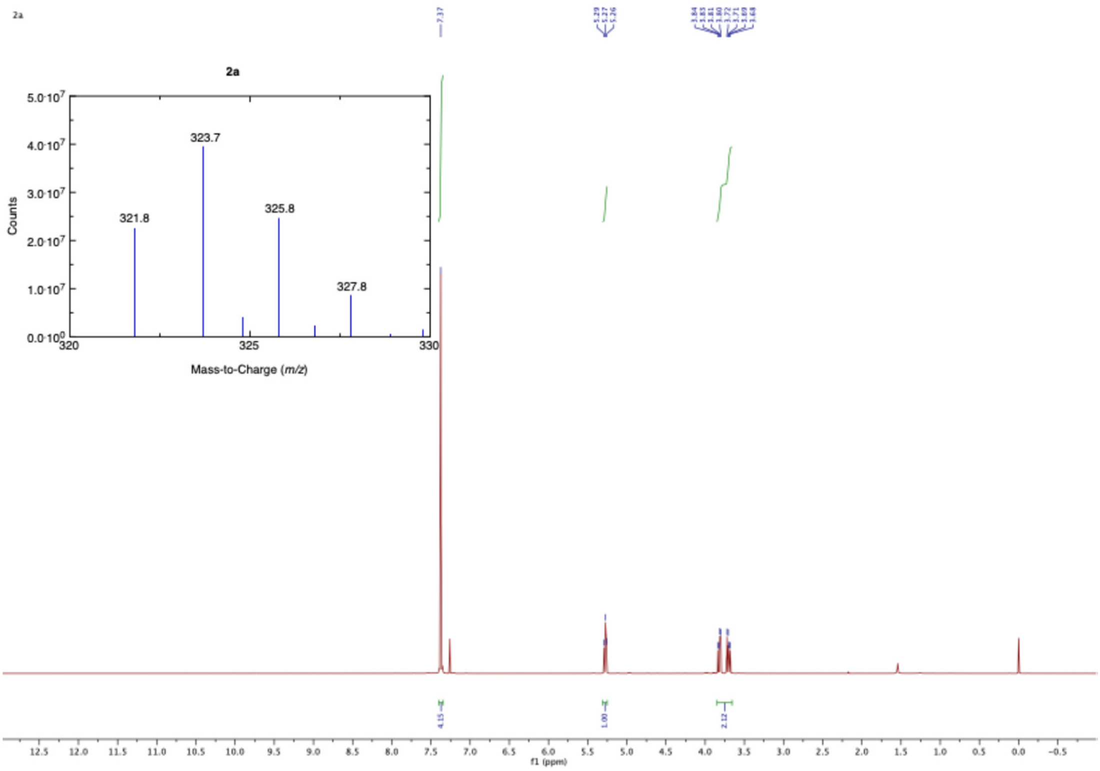
2a
7.37
5.29
5.27
5.26
2a
323.7
321.8
325.8
327.8
Counts
Mass-to-Charge (m/z)
4.15
1.00
2.12
f1 (ppm)

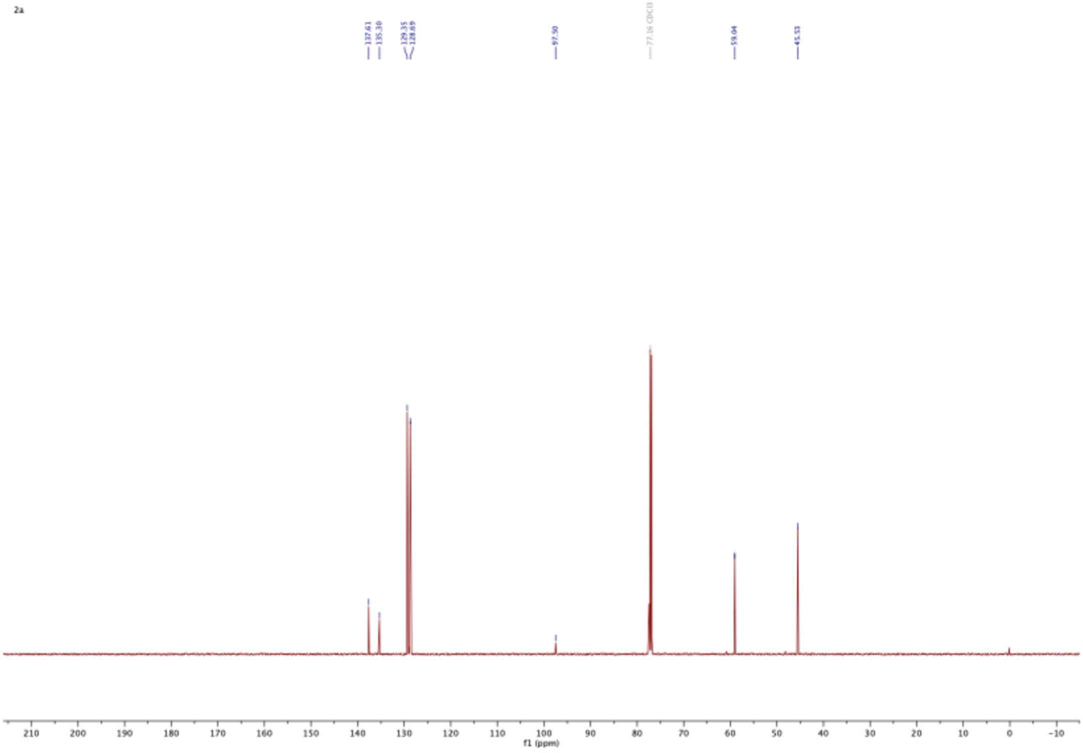
2a
137.61
135.30
129.35
128.69
97.50
77.16 CDCl3
59.04
45.53
f1 (ppm)

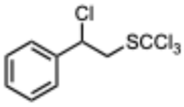
Cl
SCCl3

2b

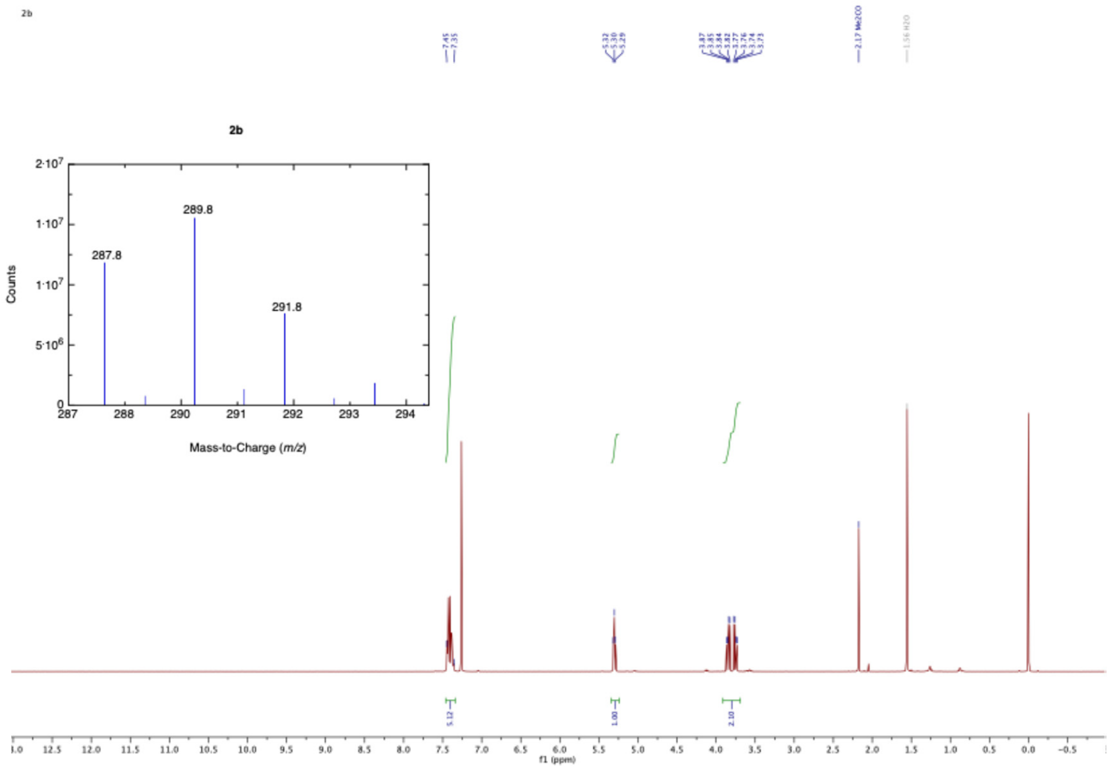
2b
7.45
7.35
5.32
5.30
5.29
2.17 Me2CO
1.56 H2O
2b
Counts
287.8
289.8
291.8
Mass-to-Charge (m/z)
5.12
1.00
2.10
f1 (ppm)

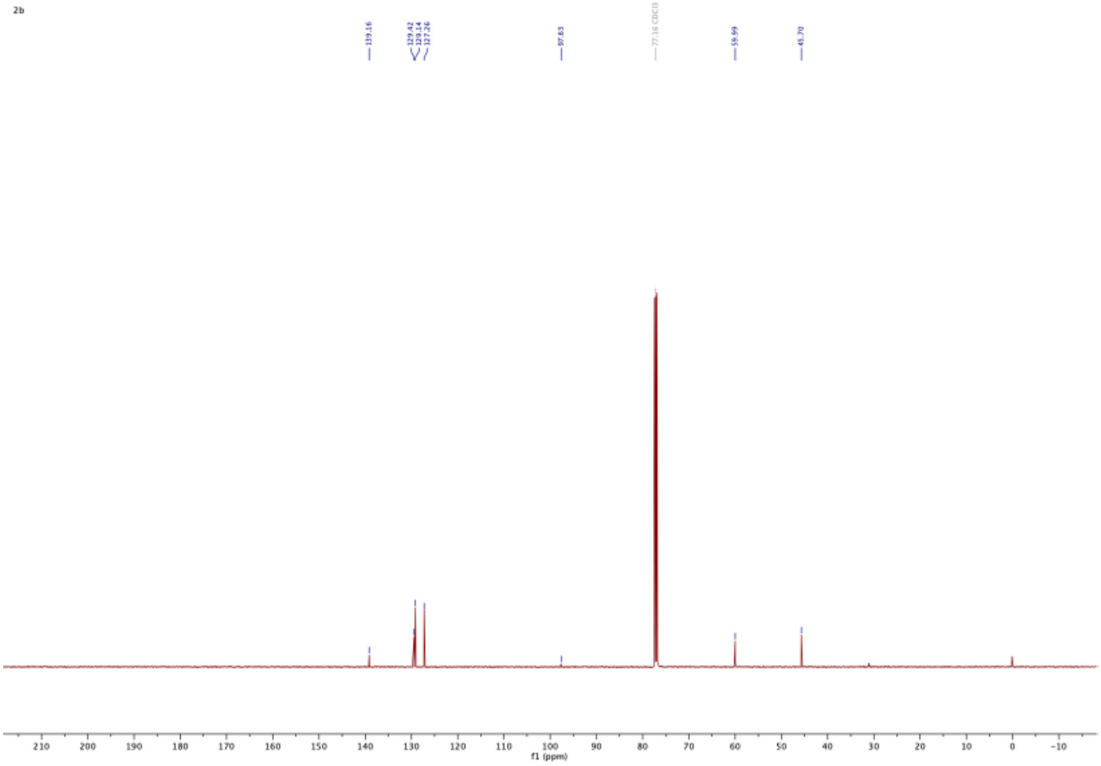
2b
139.16
129.42
129.14
127.26
97.83
77.16 CDCl3
59.99
45.70
f1 (ppm)

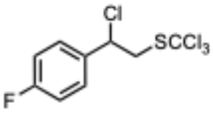

2c

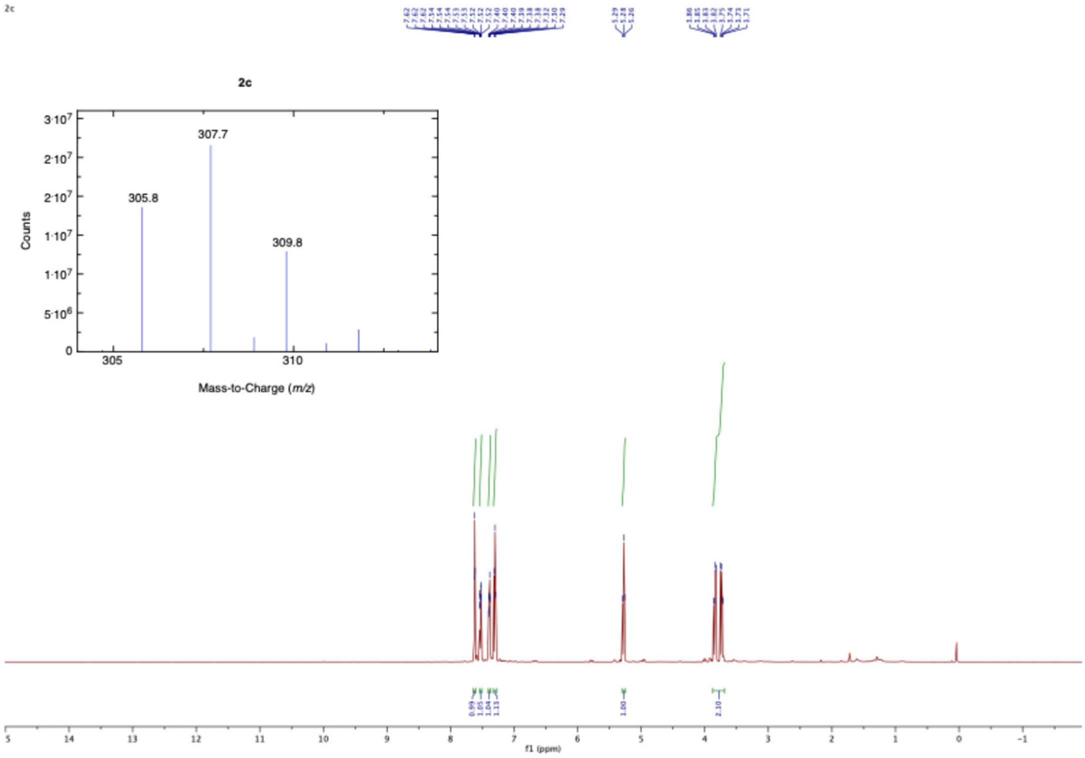
2c
2c
307.7
305.8
309.8
Counts
Mass-to-Charge (m/z)
f1 (ppm)

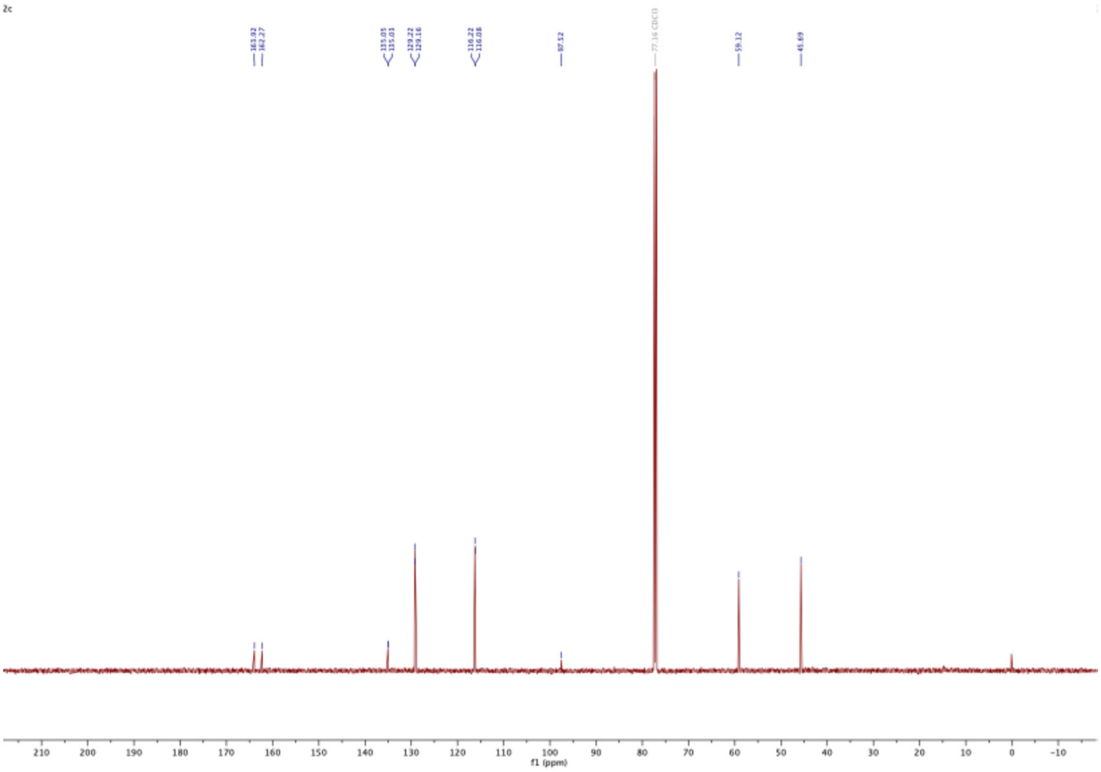
2c
163.92
162.27
135.05
135.01
129.22
129.16
116.22
116.08
97.52
77.16 CDCl3
59.12
45.69
f1 (ppm)

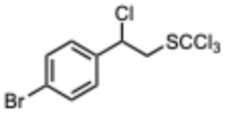
Cl
SCCl3
Br

2d

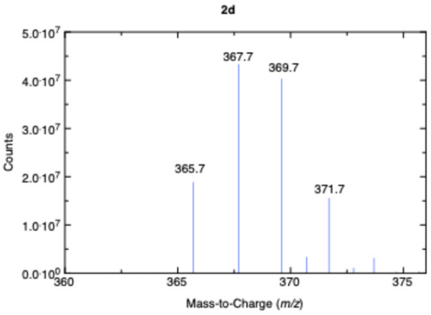
2d
Counts
Mass-to-Charge (m/z)
367.7
369.7
365.7
371.7

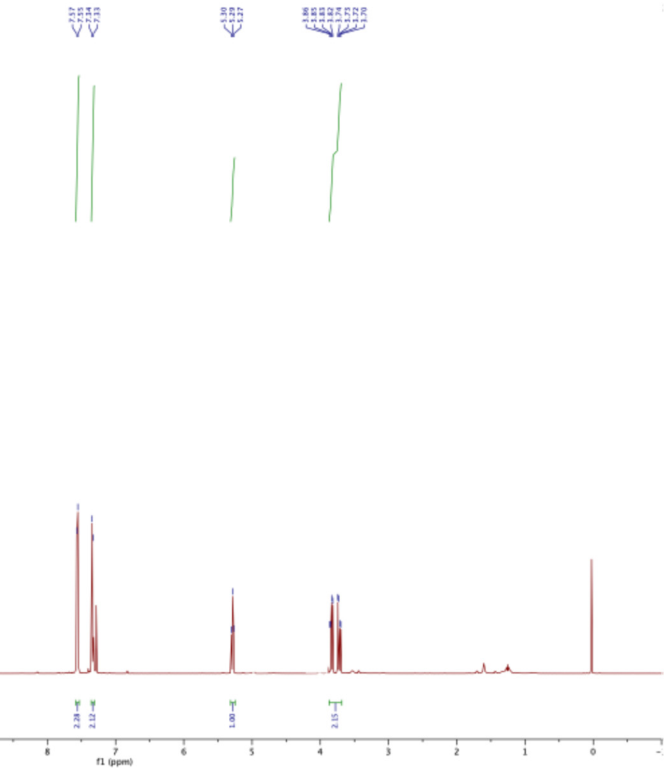
2d
7.57
7.55
7.34
7.33
5.30
5.29
5.27
3.86
3.85
3.83
3.82
3.74
3.73
3.72
3.70
2.28
2.12
1.00
2.15
f1 (ppm)

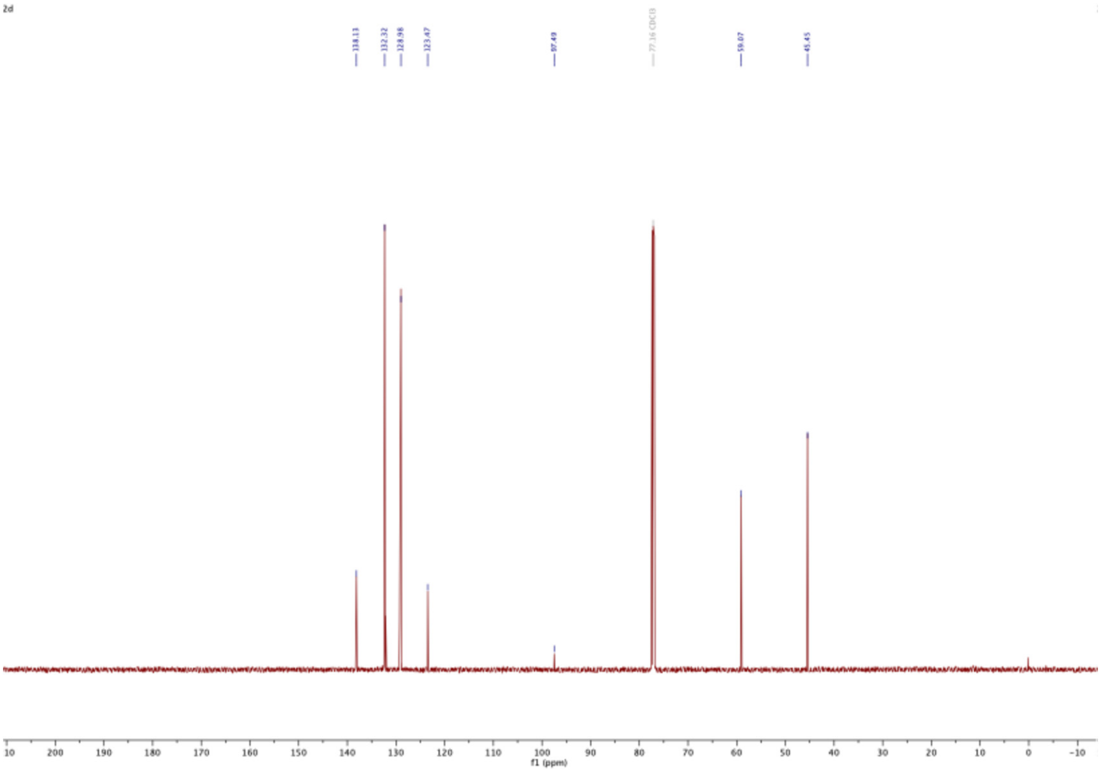
2d
138.13
132.32
128.98
123.47
97.49
77.16 CDCl3
59.07
45.45
f1 (ppm)

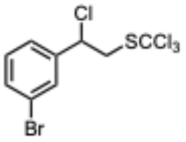
Cl
SCCl3
Br

2e

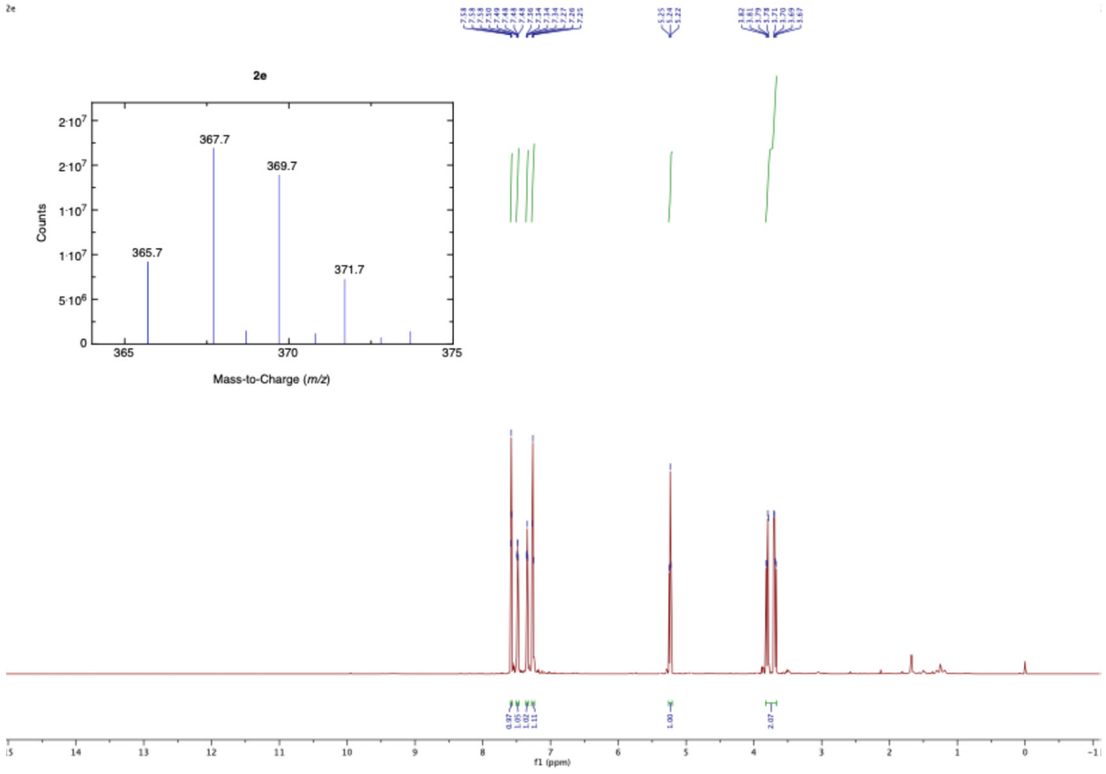
2e
367.7
369.7
365.7
371.7
Counts
Mass-to-Charge (m/z)
0.97
1.05
1.02
1.11
1.00
2.07
f1 (ppm)

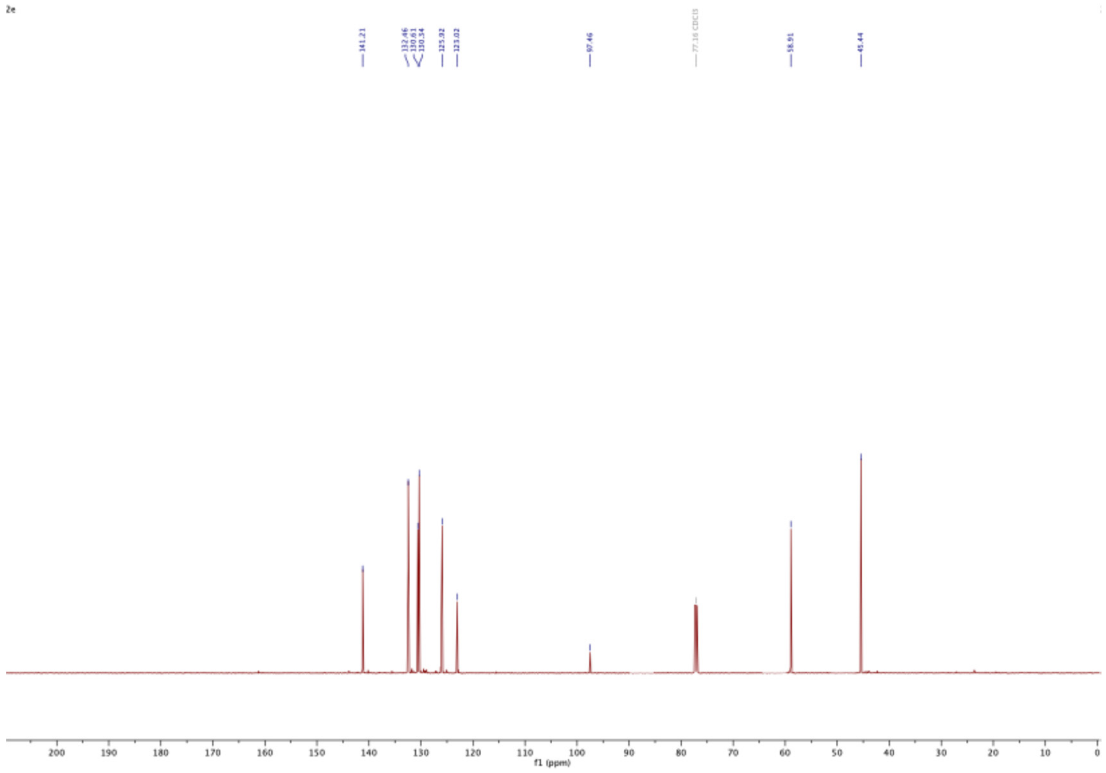
2e
141.21
132.46
130.61
130.34
125.92
123.02
97.46
77.16 CDCl3
58.91
45.44
f1 (ppm)

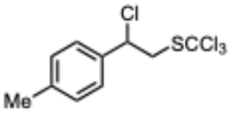
Cl
SCCl3
Me

2f

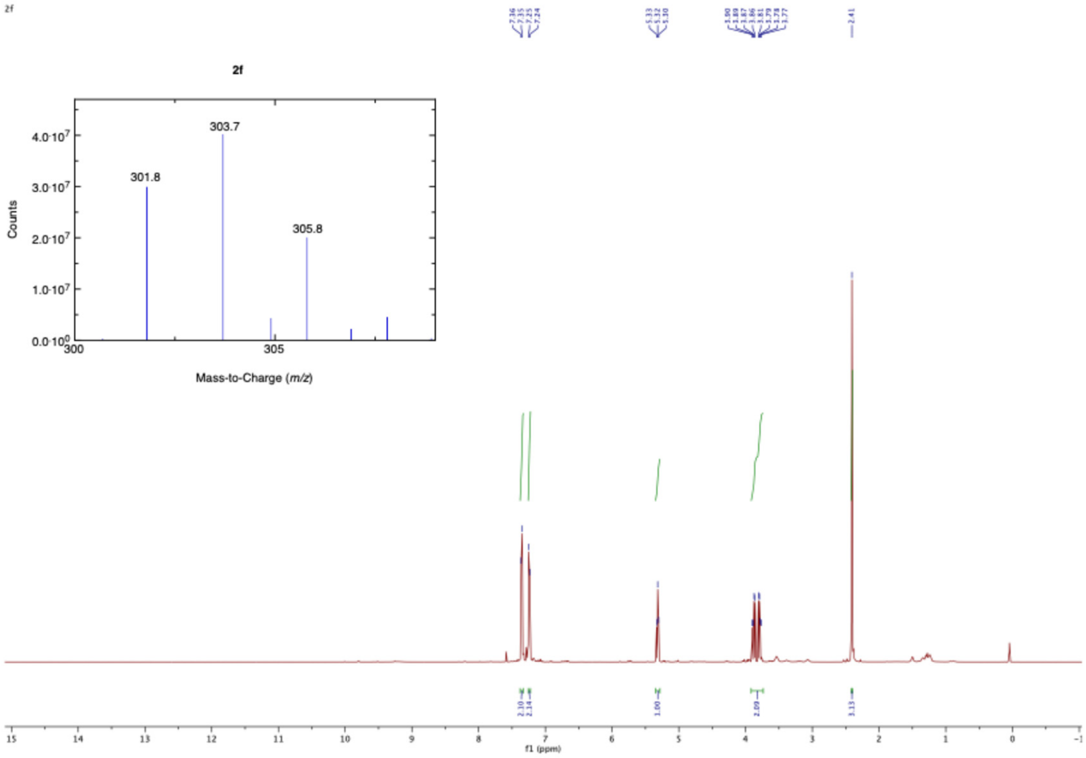
2f
2f
4.0·10^7
3.0·10^7
2.0·10^7
1.0·10^7
0.0·10^0
Counts
303.7
301.8
305.8
300
305
Mass-to-Charge (m/z)
2.10
2.14
1.00
2.09
3.13
f1 (ppm)

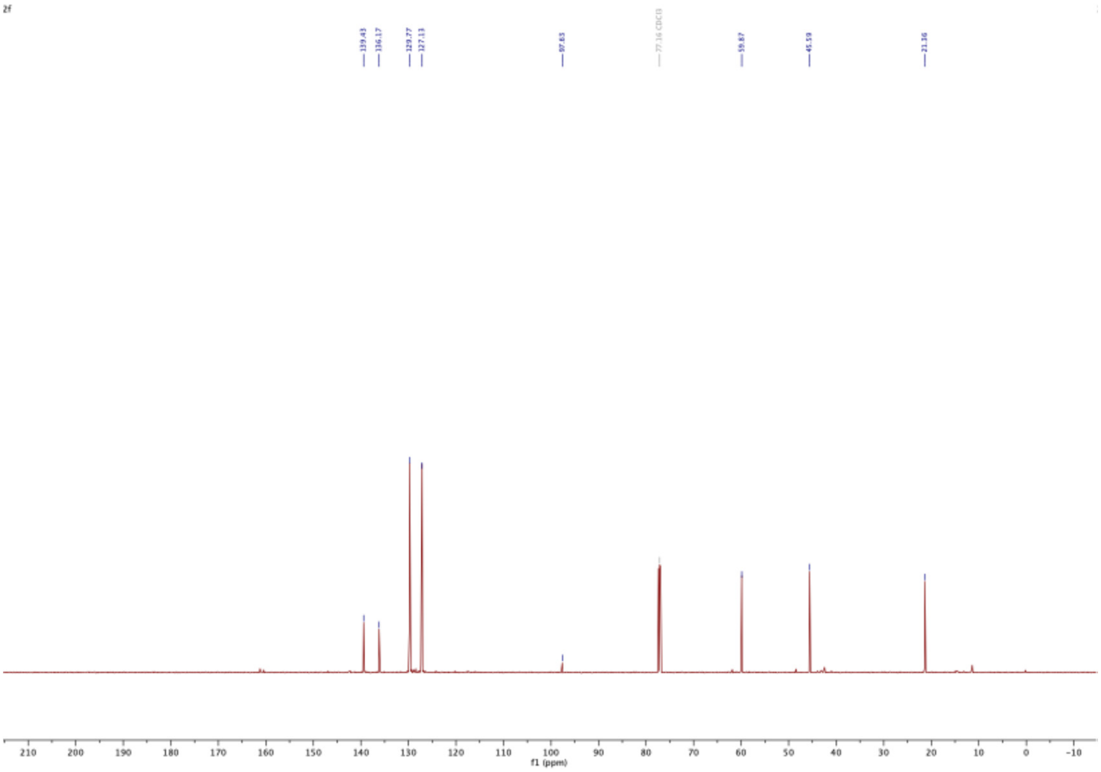
2f
139.43
136.17
129.77
127.13
97.63
77.16 CDCl3
59.87
45.59
21.16
f1 (ppm)

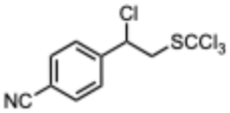

2g

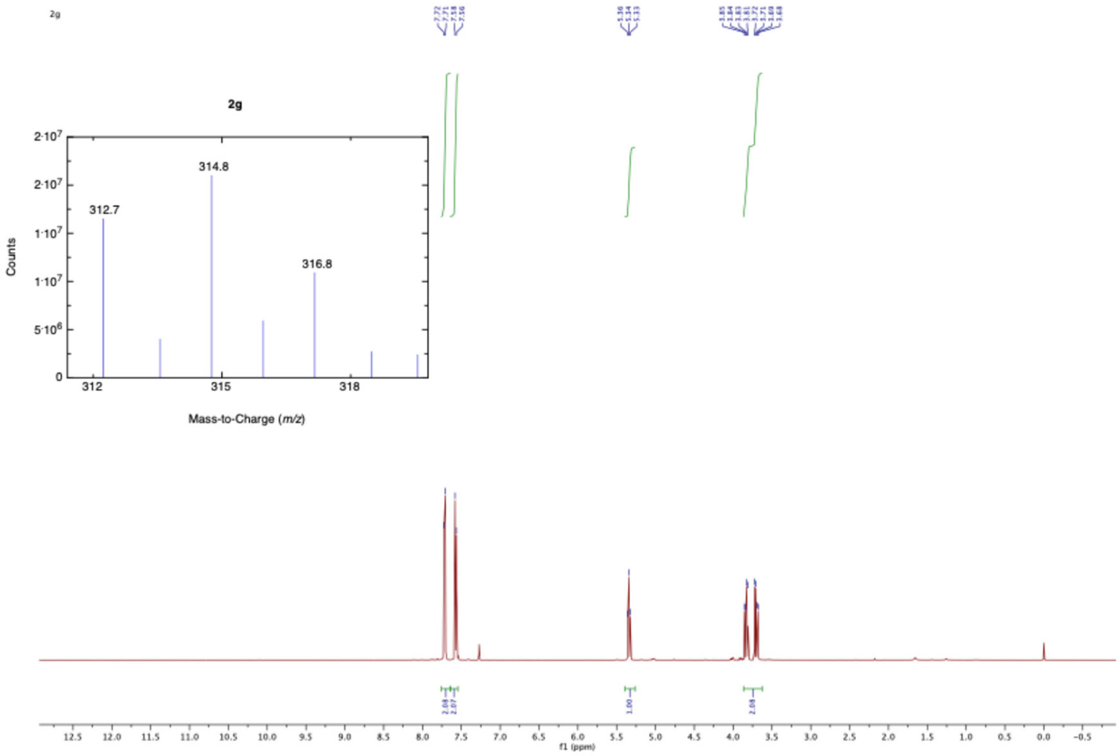
2g
2g
312.7
314.8
316.8
Counts
Mass-to-Charge (m/z)
f1 (ppm)

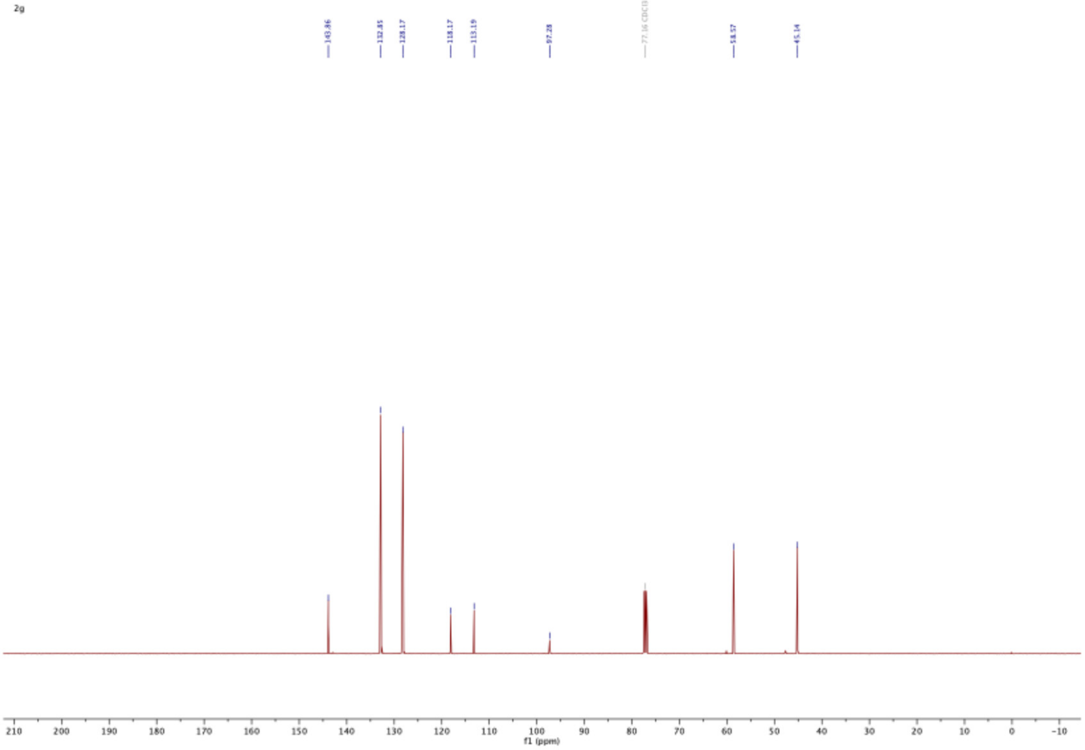
2g
143.86
132.85
128.17
118.17
113.19
97.28
77.16 CDCl3
58.57
45.14
f1 (ppm)

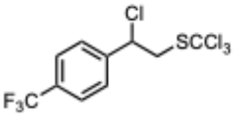
Cl
SCCl3
F3C

2h

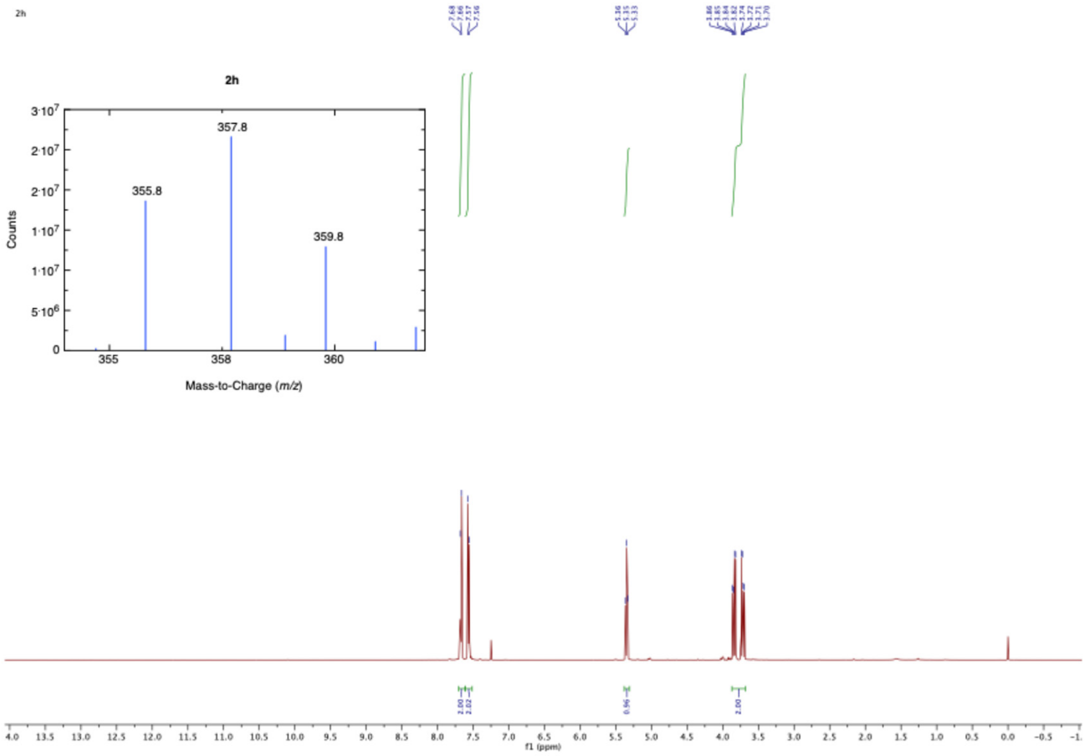
2h
2h
355.8
357.8
359.8
Counts
Mass-to-Charge (m/z)
f1 (ppm)

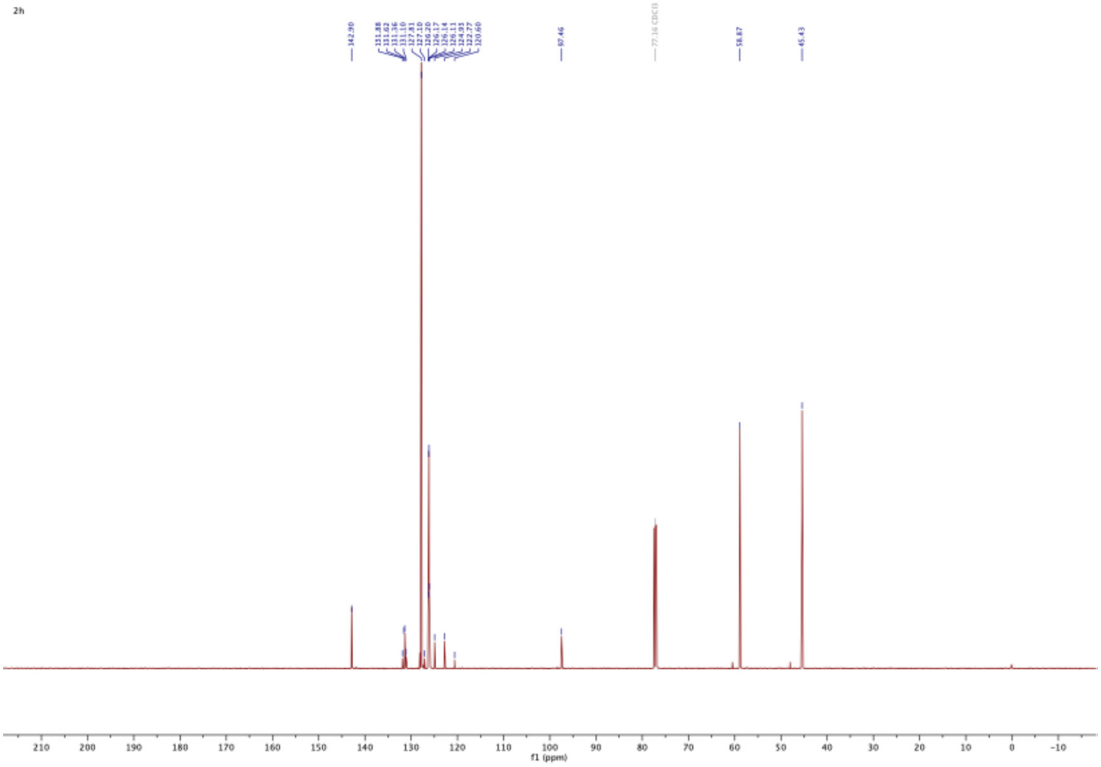
2h
142.90
97.46
77.16 CDCl3
58.87
45.43
f1 (ppm)

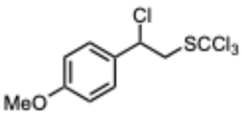
Cl
SCCl3
MeO

2i

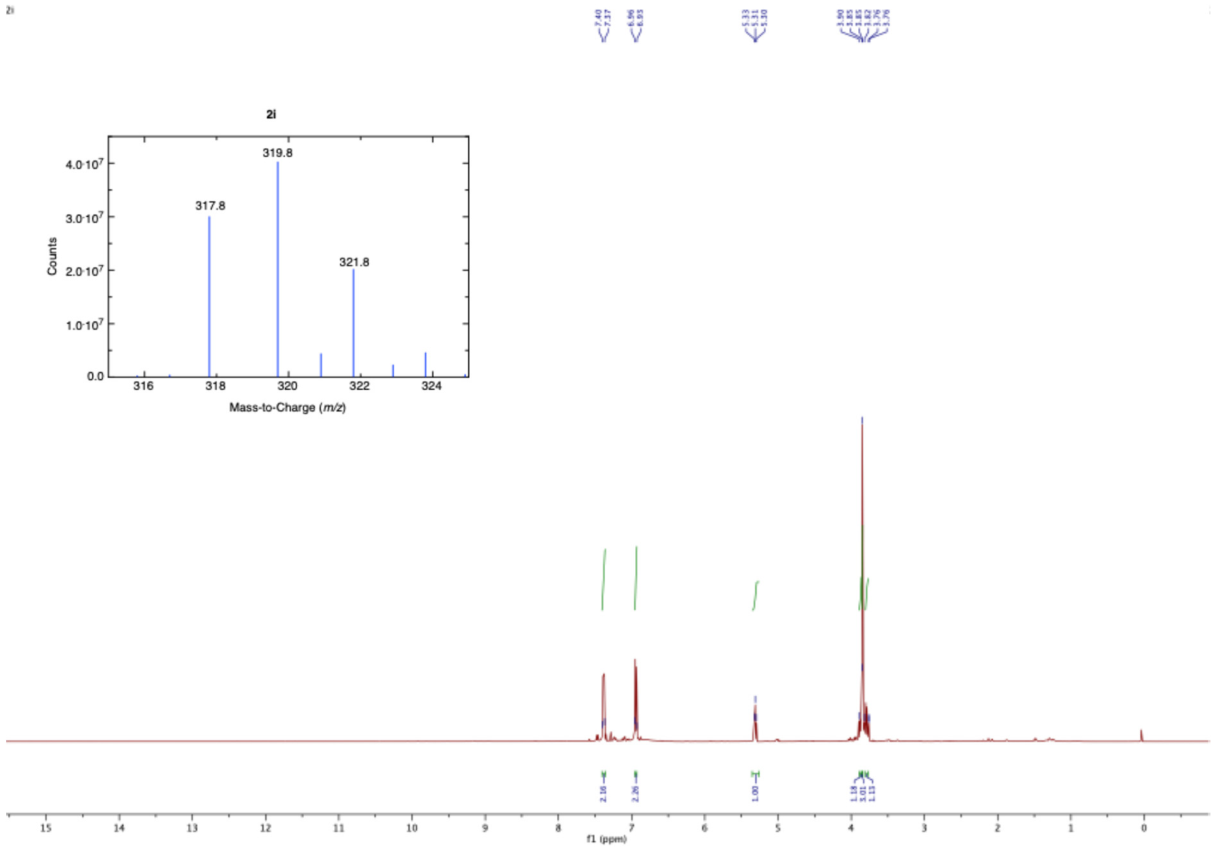
2i
7.40
7.37
6.96
6.95
5.33
5.31
5.30
2i
319.8
317.8
321.8
Counts
Mass-to-Charge (m/z)
f1 (ppm)

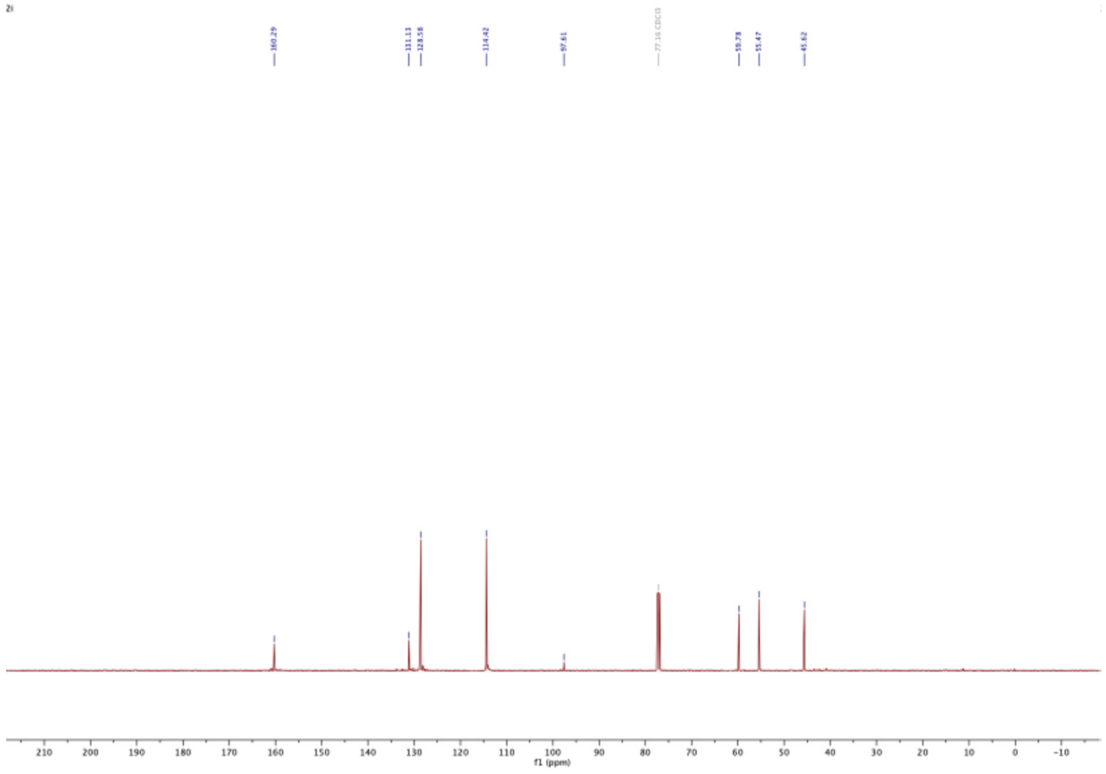
2i
160.29
131.13
128.58
114.42
97.61
77.16 CDCl3
59.78
55.47
45.62
f1 (ppm)

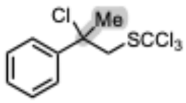
Cl
Me
SCCl3

2j

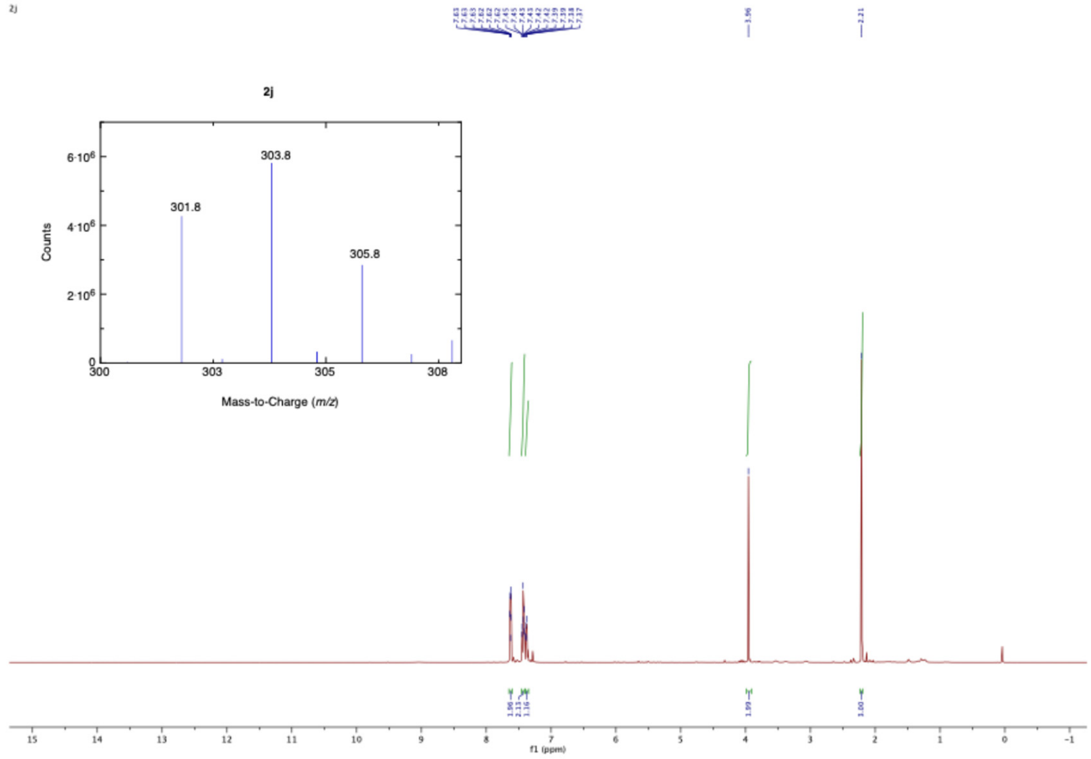
2j
3.96
2.21
2j
6·10^6
4·10^6
2·10^6
0
Counts
303.8
301.8
305.8
300
303
305
308
Mass-to-Charge (m/z)
1.96
2.13
1.16
1.99
3.00
f1 (ppm)

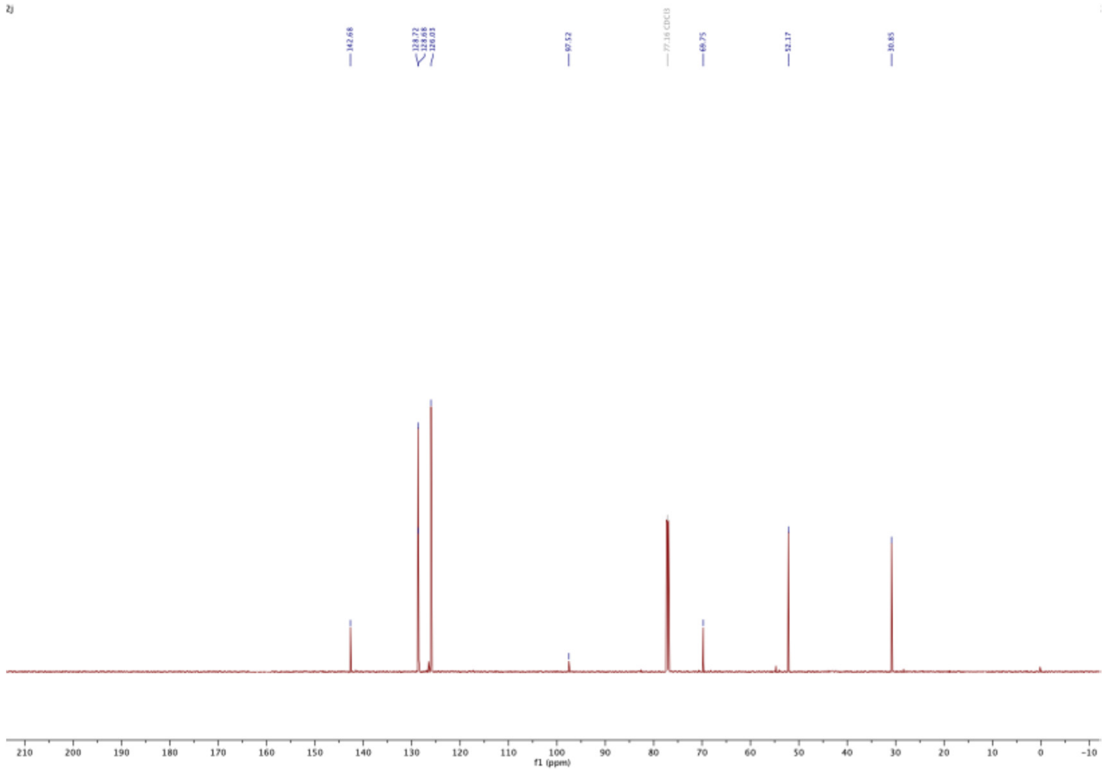
2j
142.68
128.72
128.68
126.03
97.52
77.16 CDCl3
69.75
52.17
30.85
f1 (ppm)

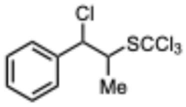

2k

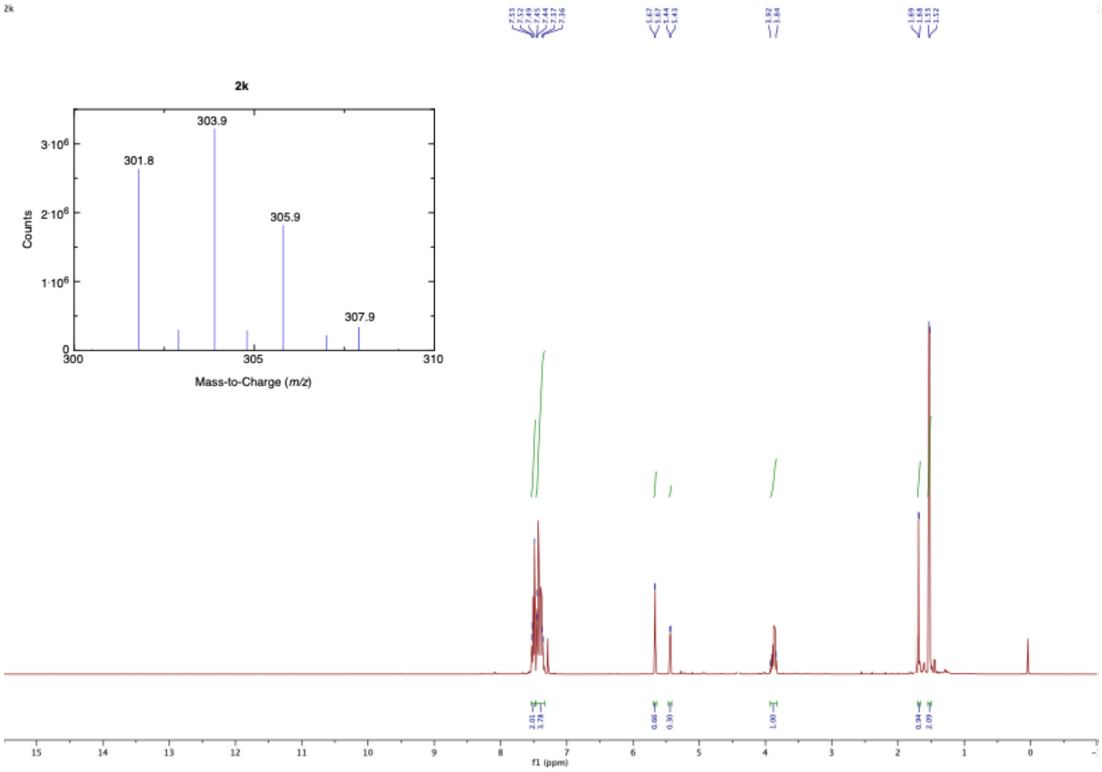

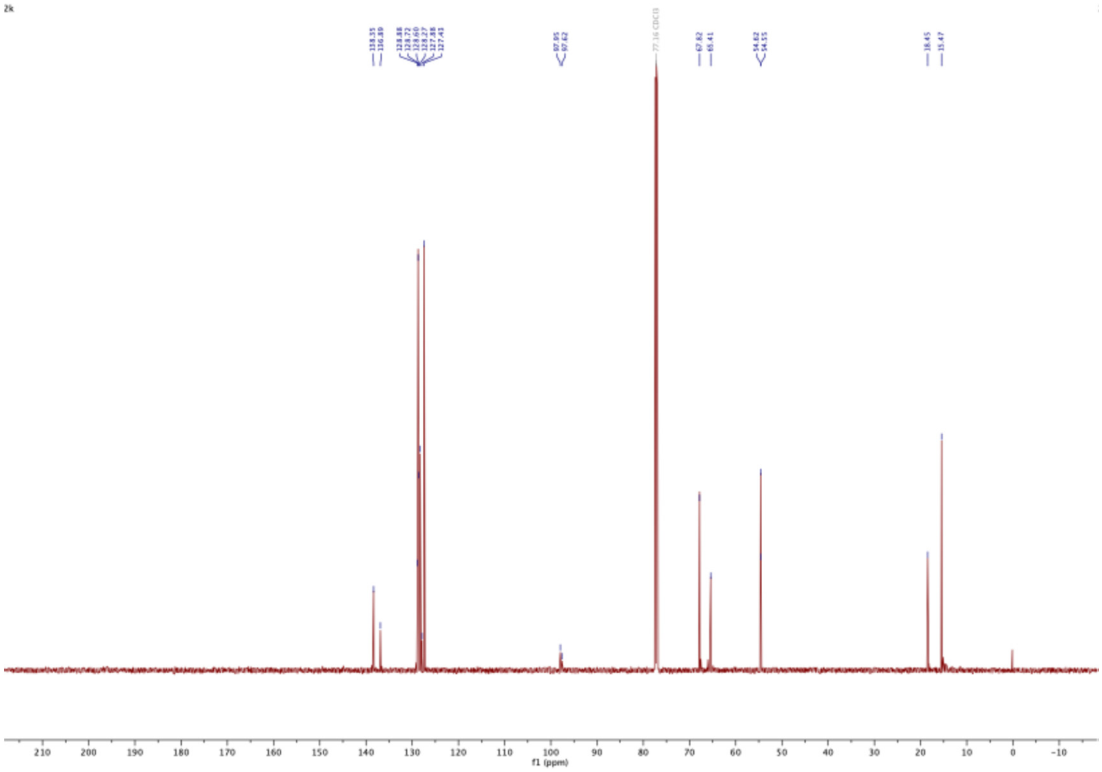

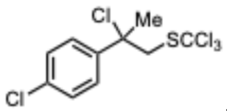
Cl
Me
SCCl3
Cl

2l

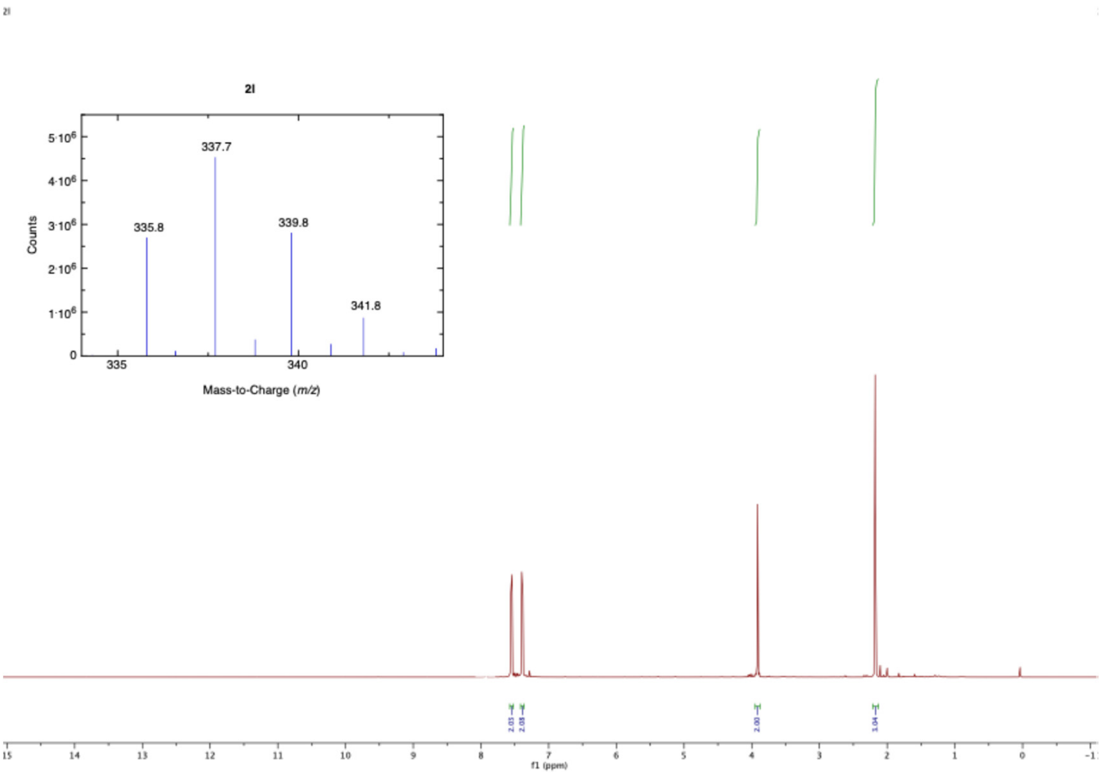
2l
2l
335.8
337.7
339.8
341.8
Counts
Mass-to-Charge (m/z)
2.03
2.08
2.00
3.04
f1 (ppm)

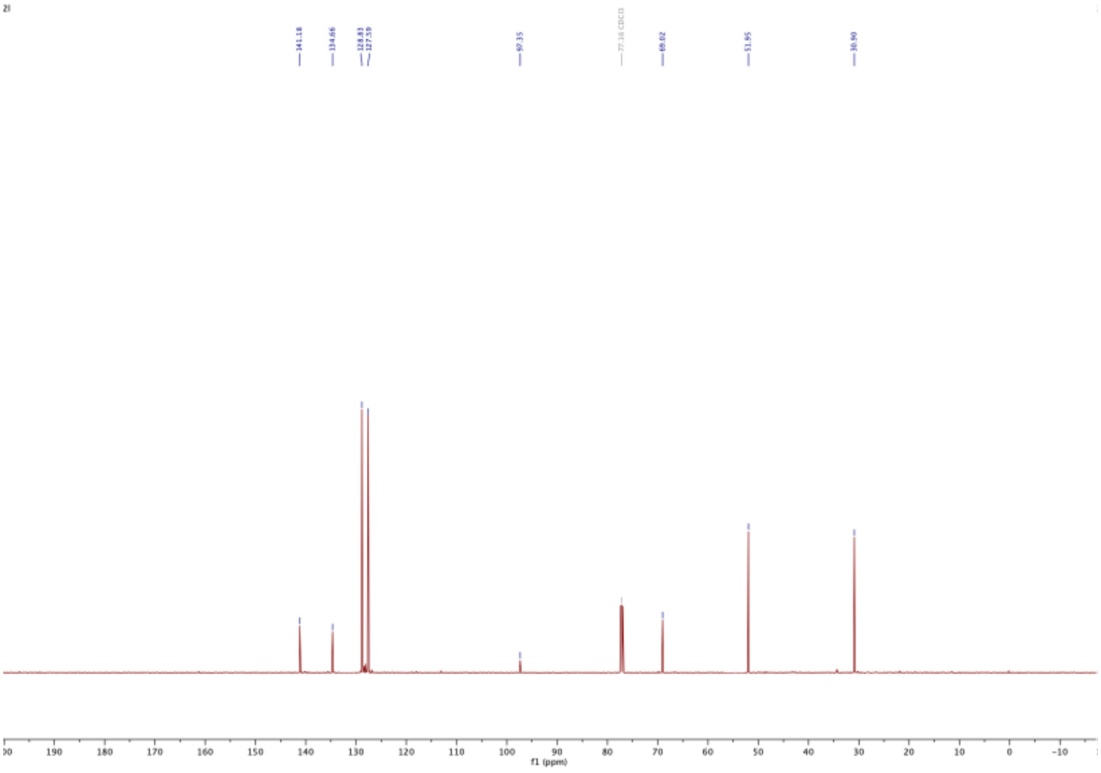
2l
141.18
134.66
128.83
127.59
97.35
77.16 CDCl3
69.02
51.95
30.90
f1 (ppm)

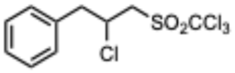

SO2CCl3
Cl

2m°

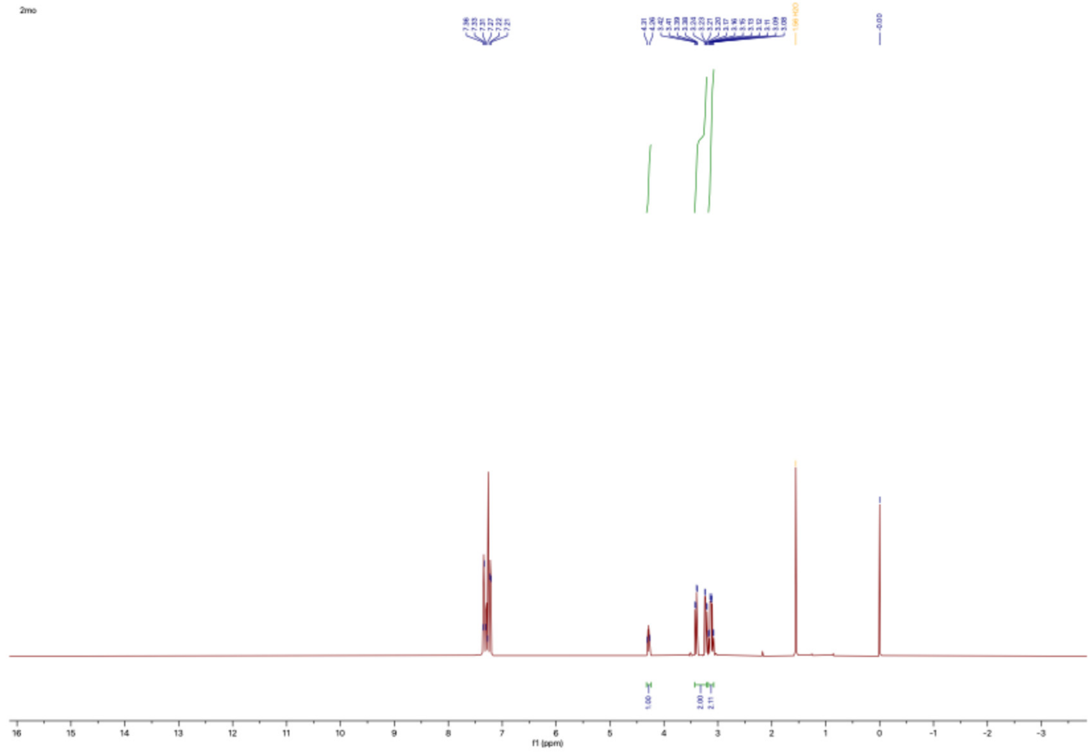

2mo
f1 (ppm)

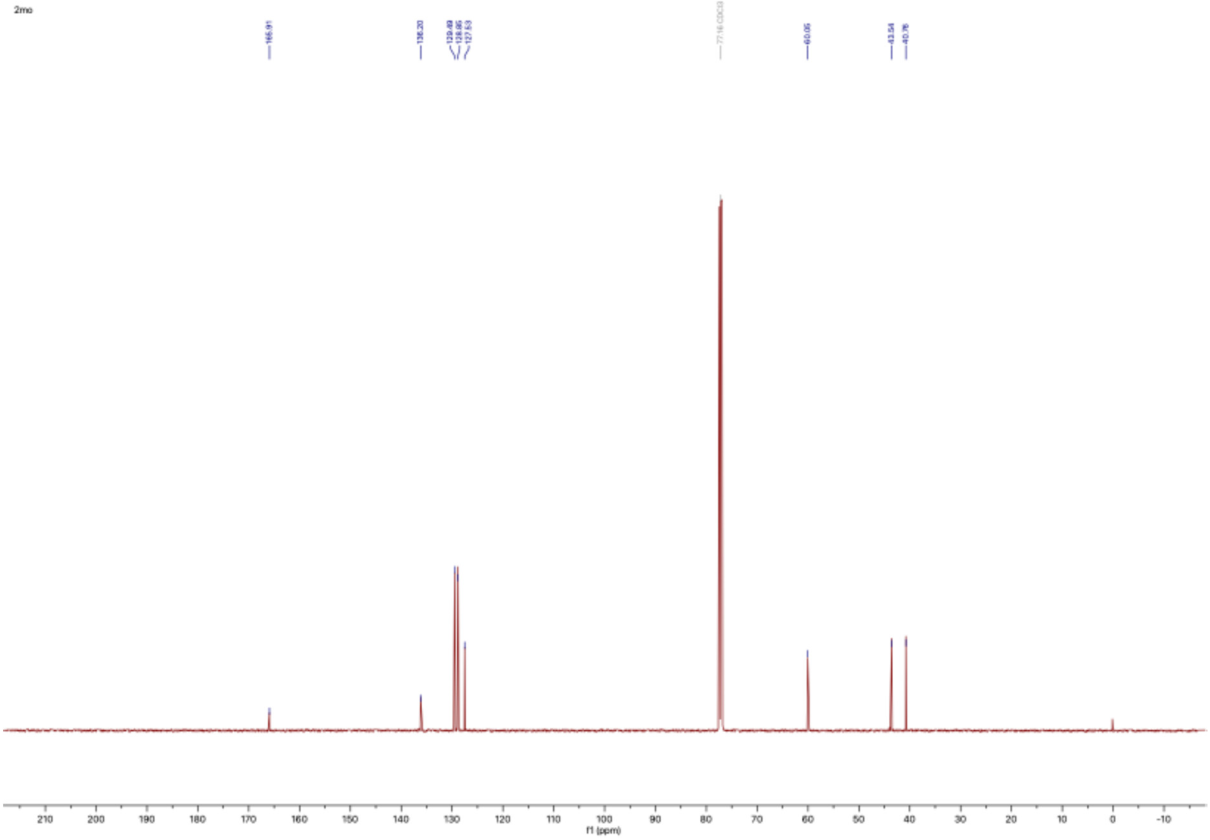

2mo
165.91
136.20
129.49
128.85
127.63
77.16 CDCl3
60.05
43.54
40.76
f1 (ppm)

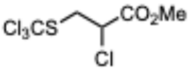
Cl3CS
CO2Me
Cl

2n

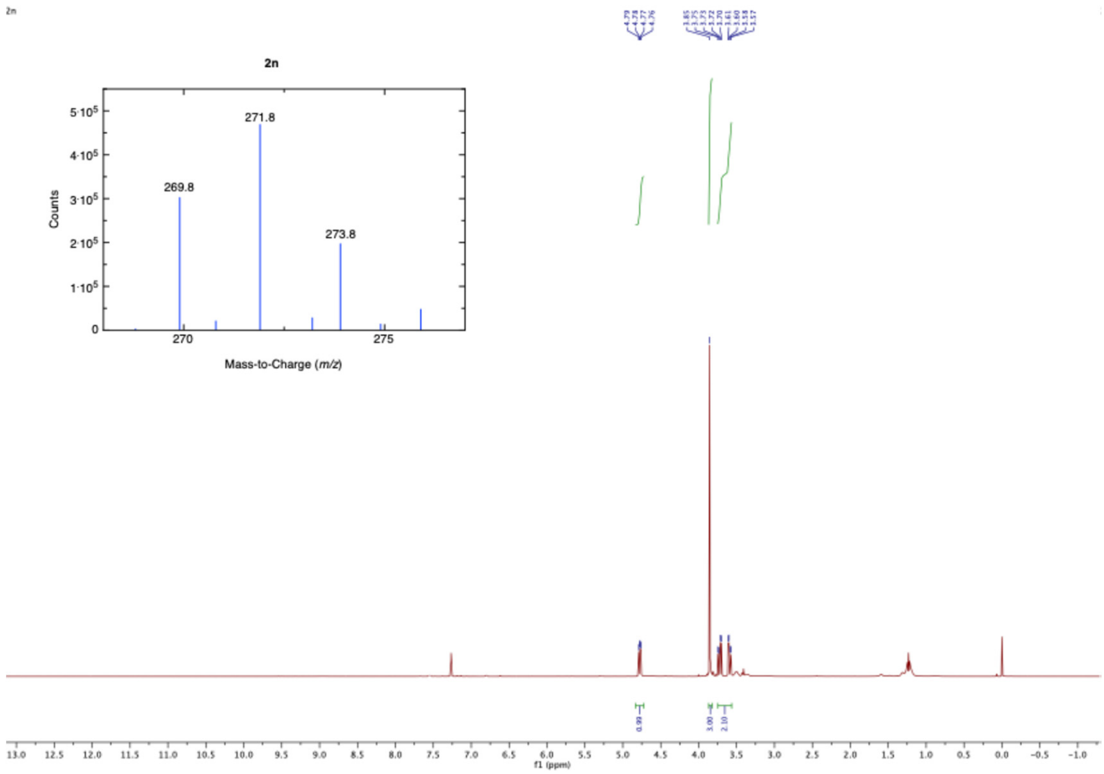
2n
2n
271.8
269.8
273.8
Counts
Mass-to-Charge (m/z)
f1 (ppm)

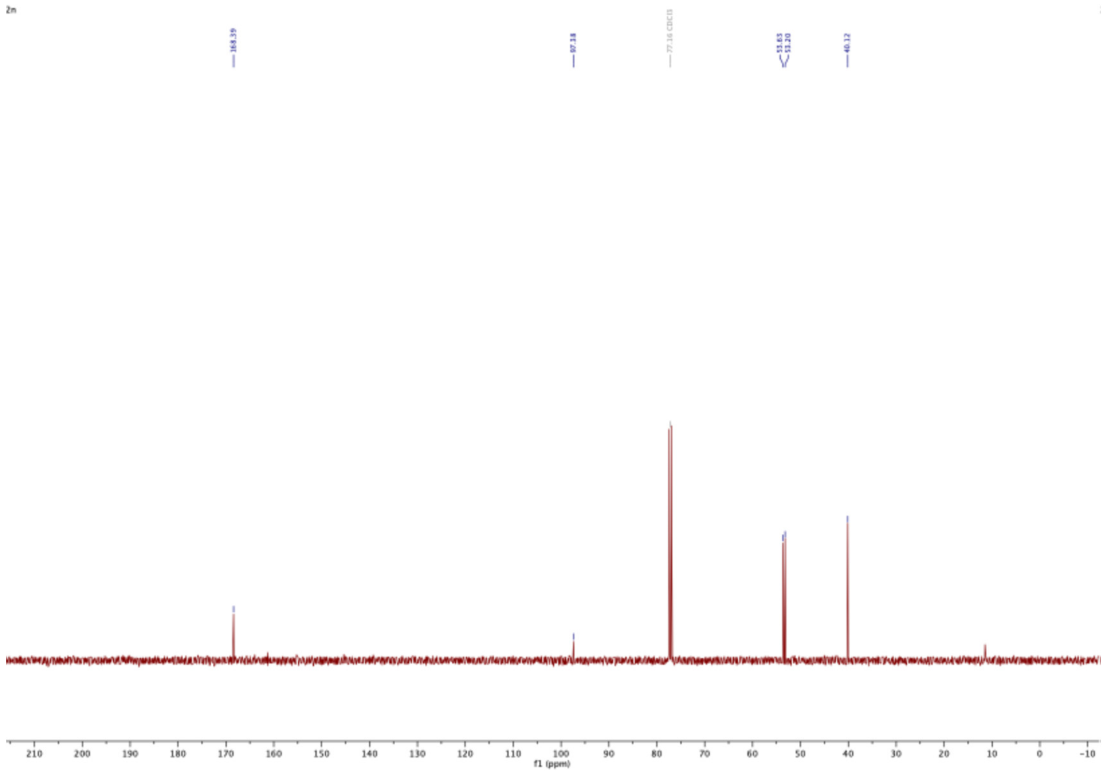
2n
168.59
97.18
77.16 CDCl3
40.12
f1 (ppm)

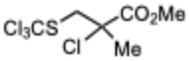
Cl3CS
CO2Me
Cl
Me

2o

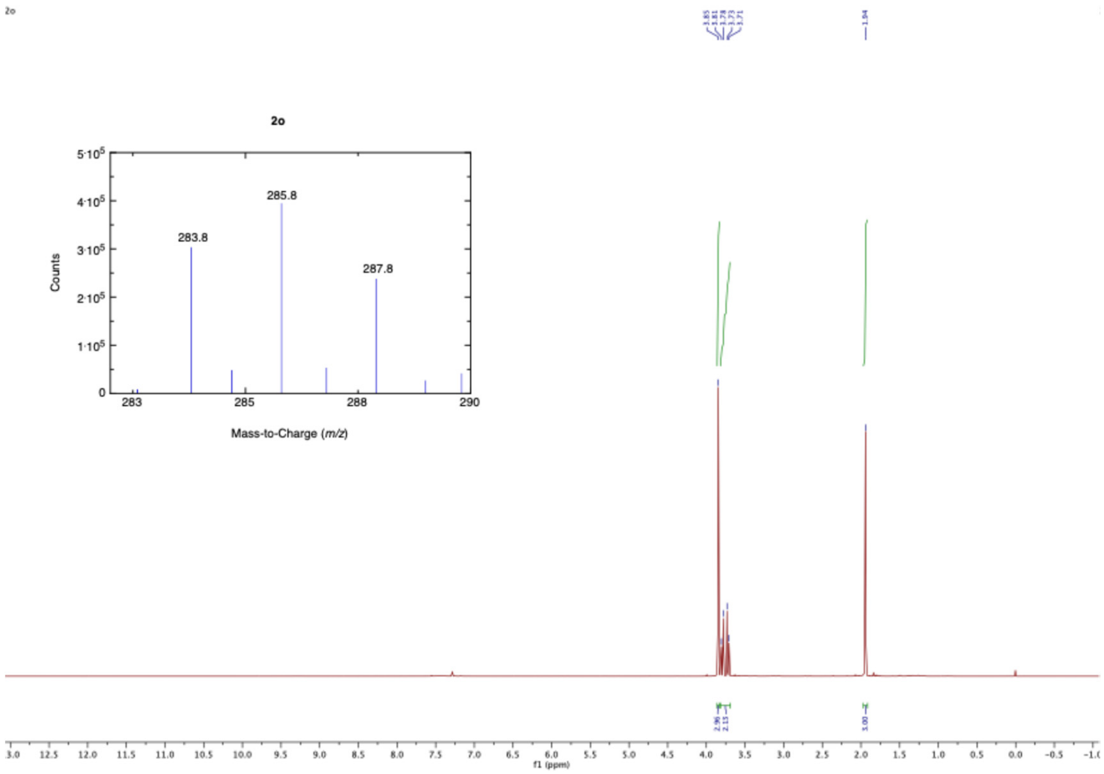
2o
1.94
2o
Counts
283.8
285.8
287.8
Mass-to-Charge (m/z)
2.96
2.13
3.00
f1 (ppm)

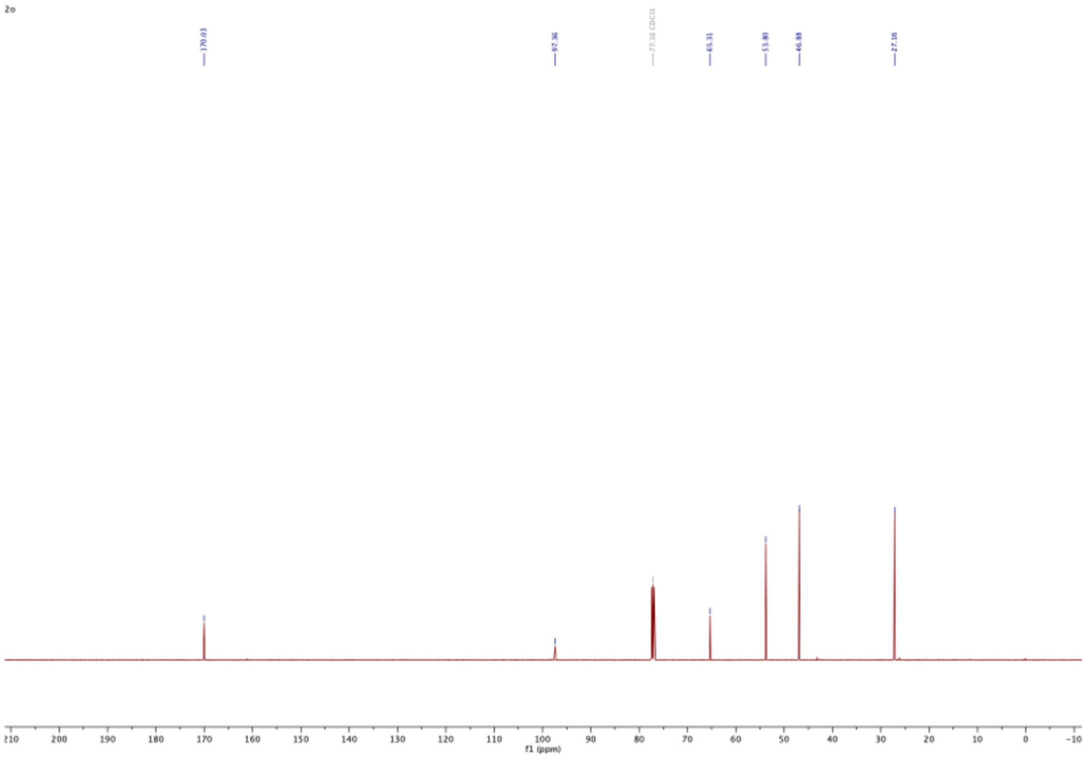
2o
170.03
97.36
77.16 CDCl3
65.31
53.80
46.88
27.10
f1 (ppm)

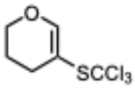
O
SCCl3
2p

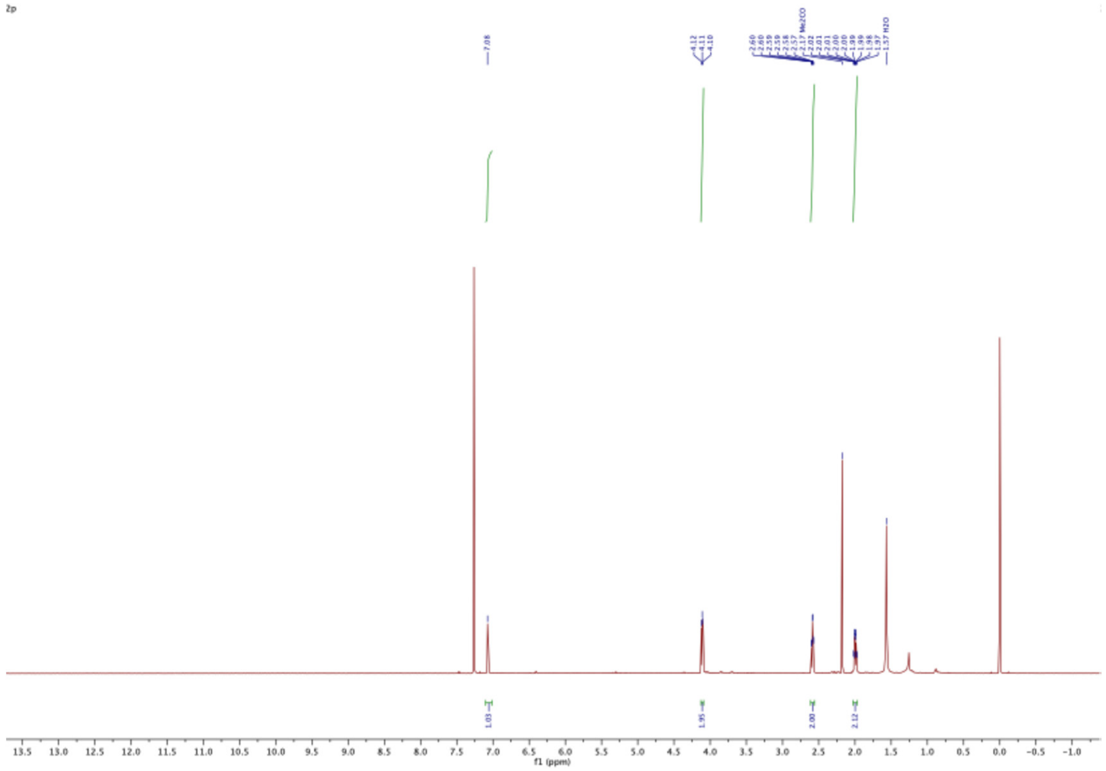
2p
7.08
4.12
4.11
4.10
2.60
2.60
2.59
2.59
2.58
2.57
2.17 Me2CO
2.02
2.01
2.01
2.00
2.00
1.99
1.99
1.98
1.97
1.57 H2O
1.03
1.95
2.00
2.12
13.5 13.0 12.5 12.0 11.5 11.0 10.5 10.0 9.5 9.0 8.5 8.0 7.5 7.0 6.5 6.0 5.5 5.0 4.5 4.0 3.5 3.0 2.5 2.0 1.5 1.0 0.5 0.0 -0.5 -1.0
f1 (ppm)

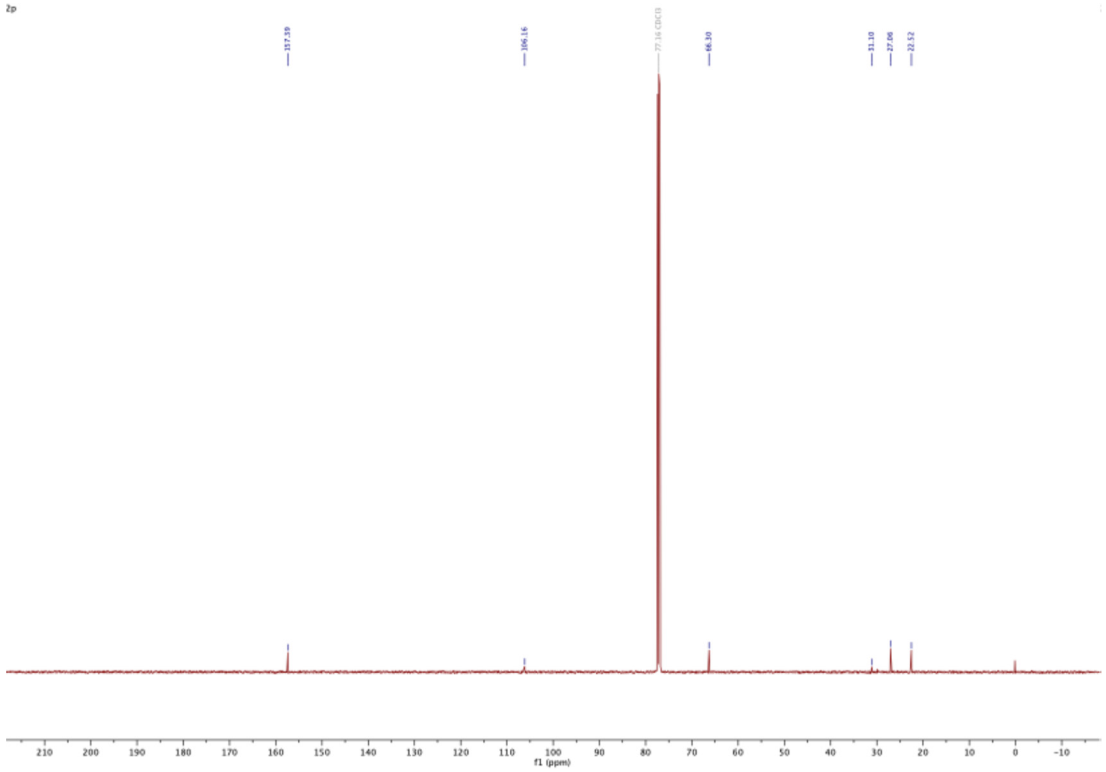
2p
157.59
106.16
77.16 CDCl3
66.30
31.10
27.06
22.52
210 200 190 180 170 160 150 140 130 120 110 100 90 80 70 60 50 40 30 20 10 0 -10
f1 (ppm)

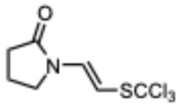
O
N
SCCl3

2r

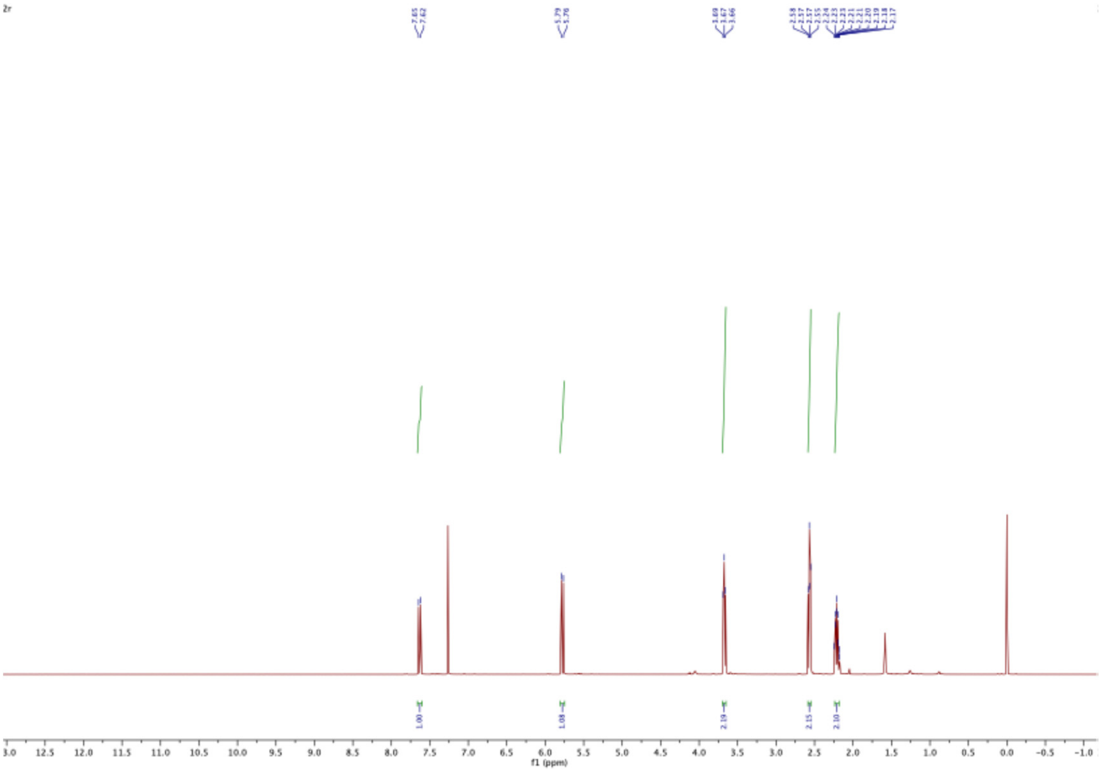
2r
7.65
7.62
5.79
5.76
1.00
1.08
2.19
2.15
2.10
f1 (ppm)

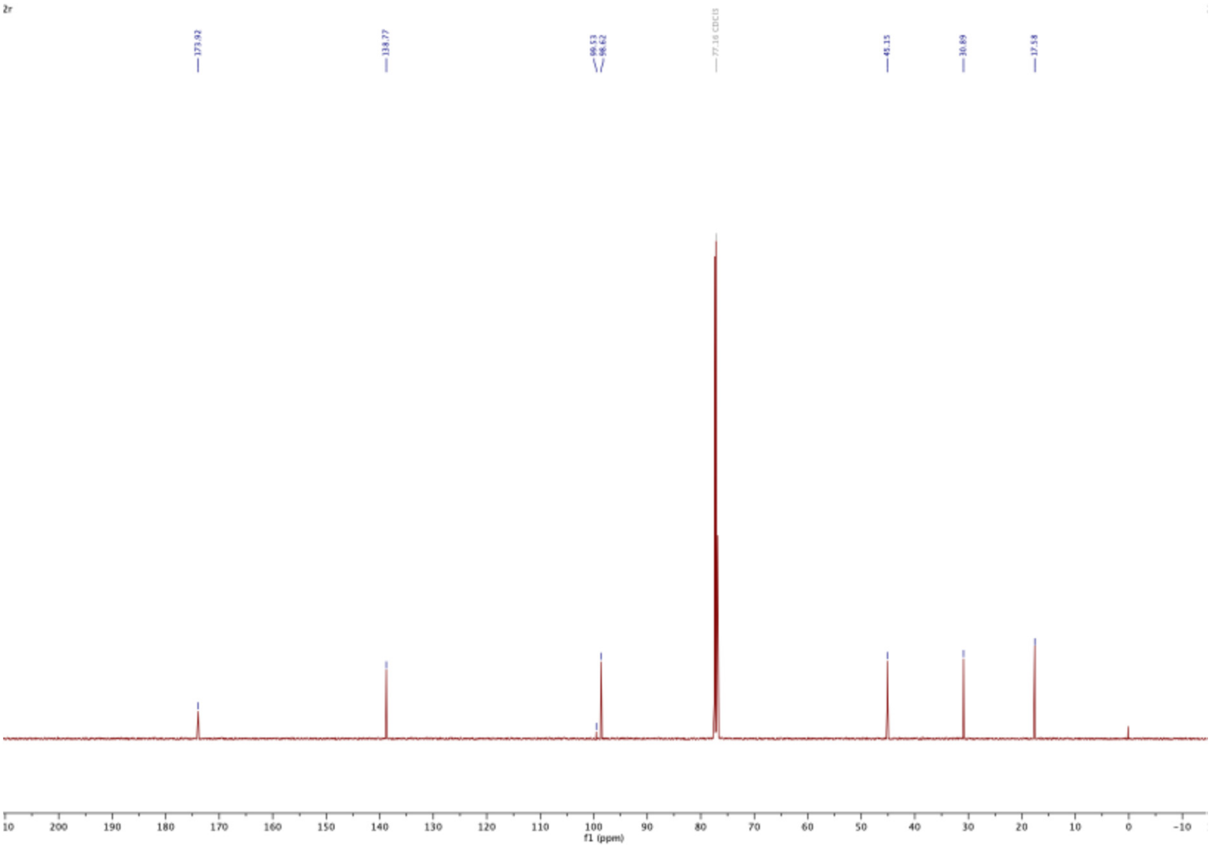
2r
173.92
138.77
99.53
98.62
77.16 CDCl3
45.15
30.89
17.58
f1 (ppm)

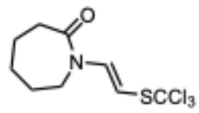

2s

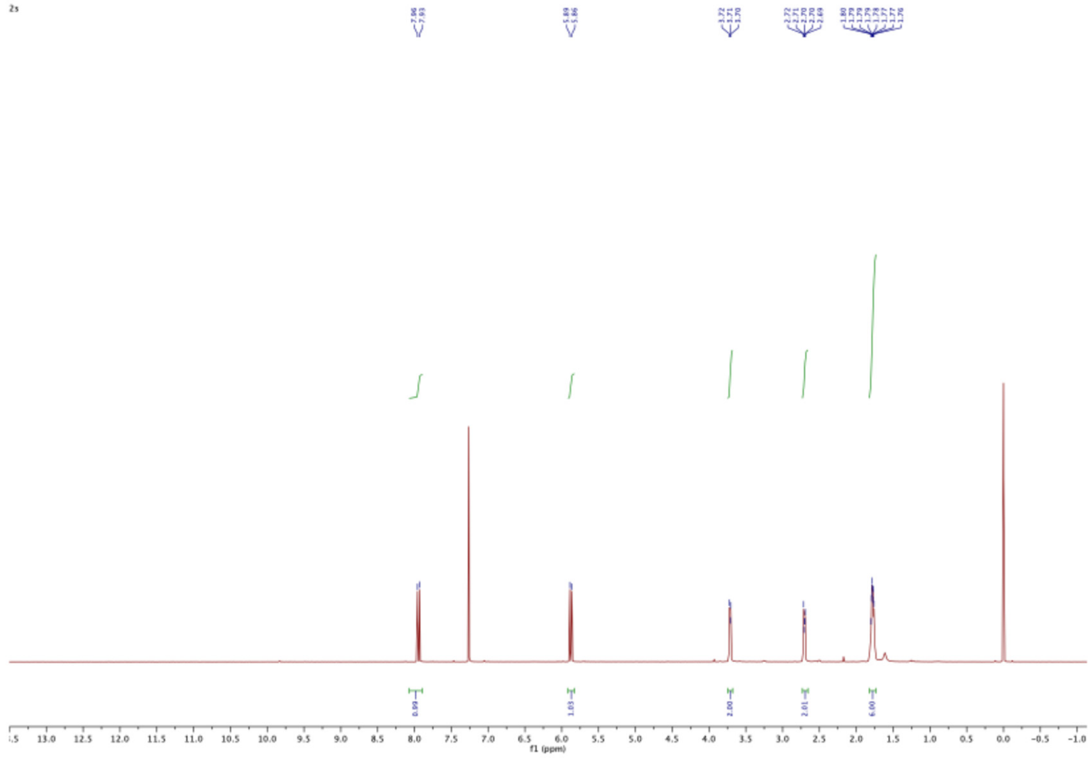

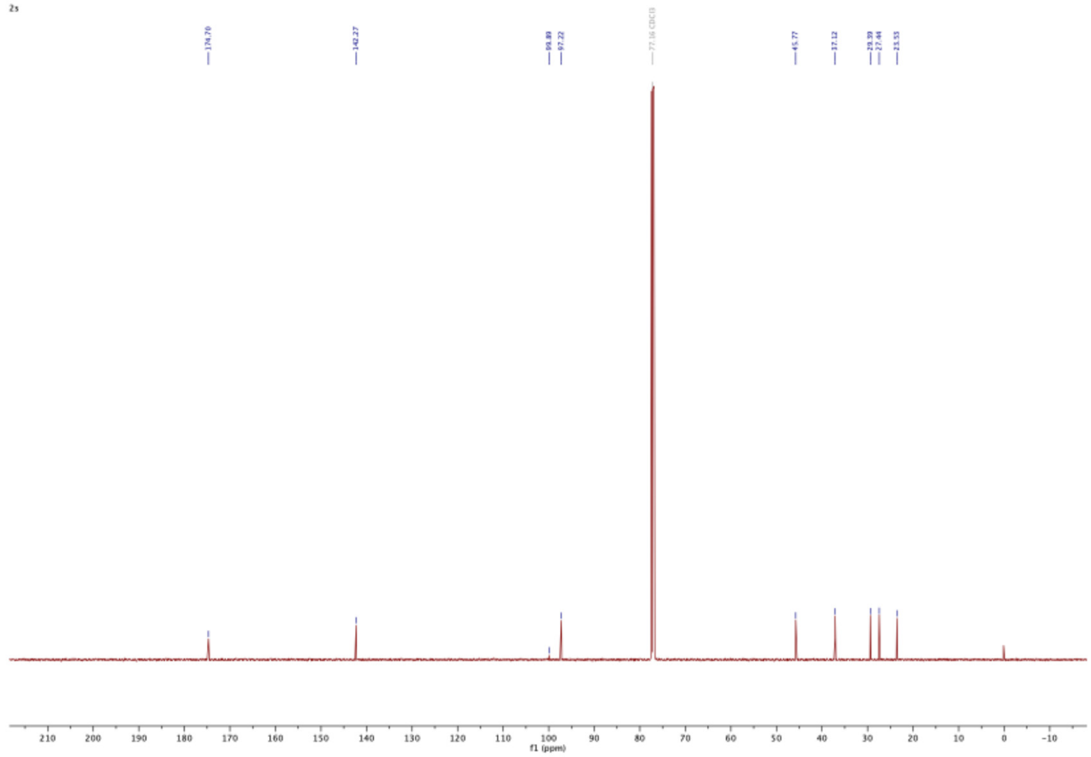

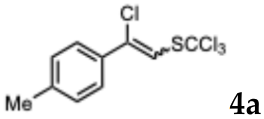
Cl
SCCl3
Me

4a

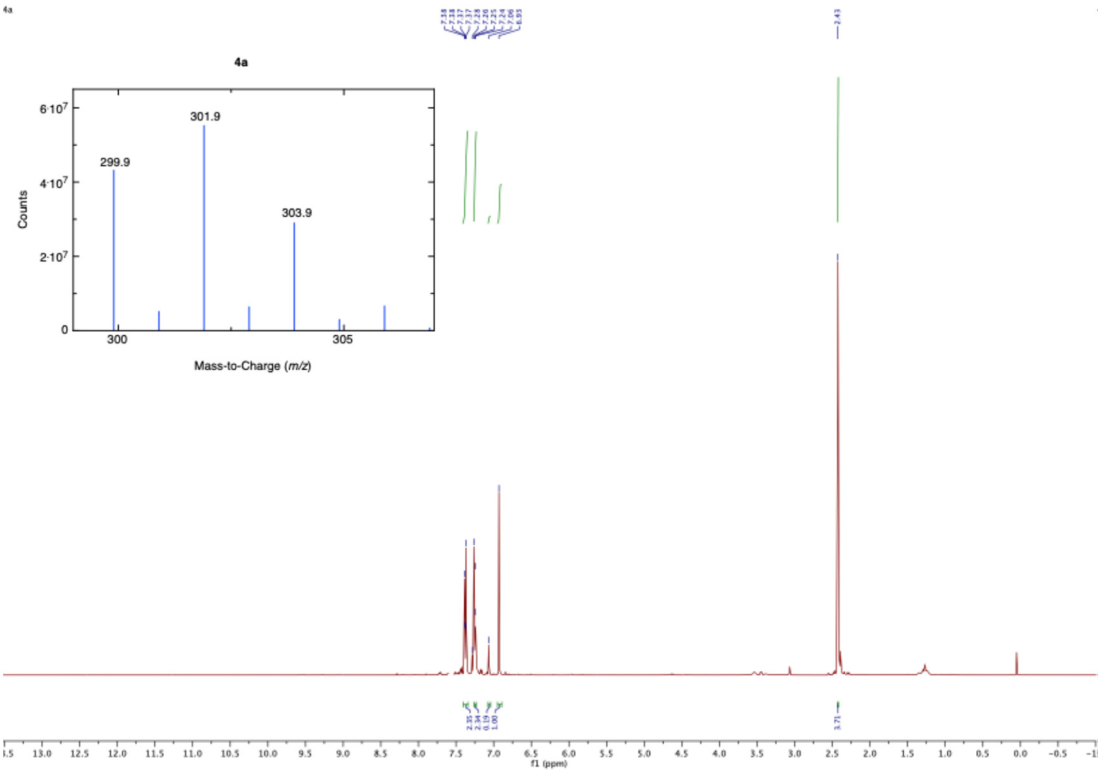
4a
4a
301.9
299.9
303.9
Counts
Mass-to-Charge (m/z)
f1 (ppm)

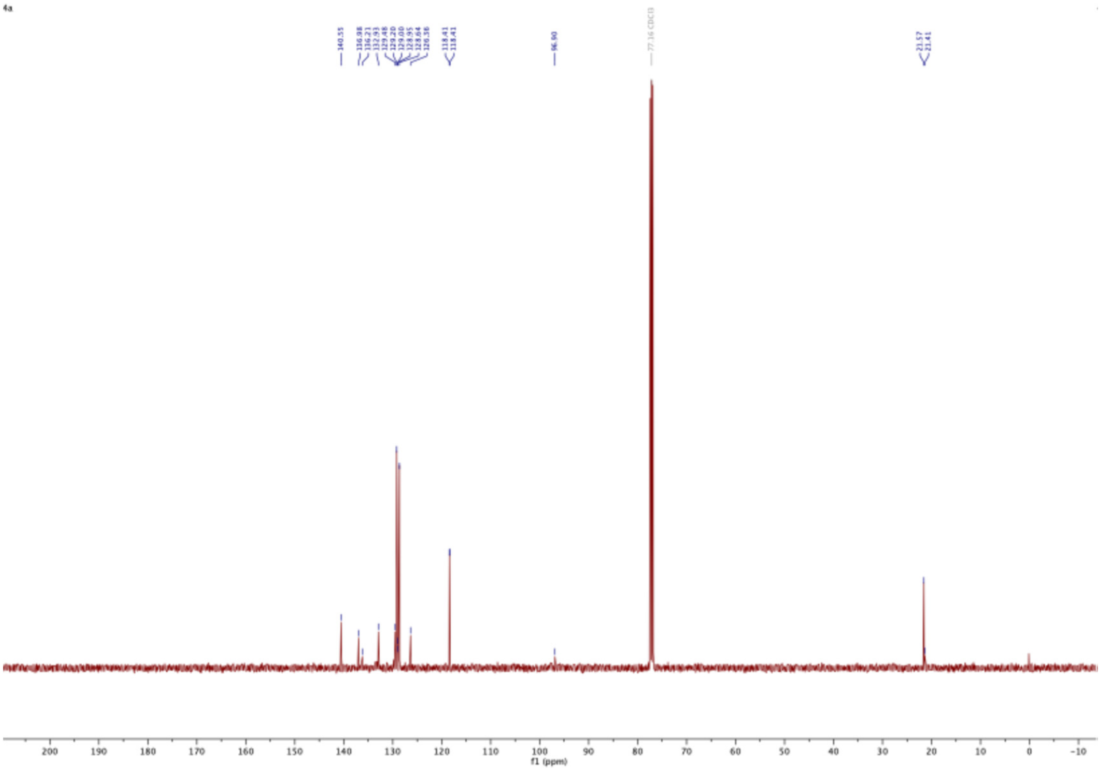
4a
f1 (ppm)

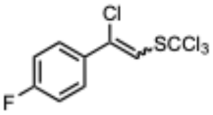

4b

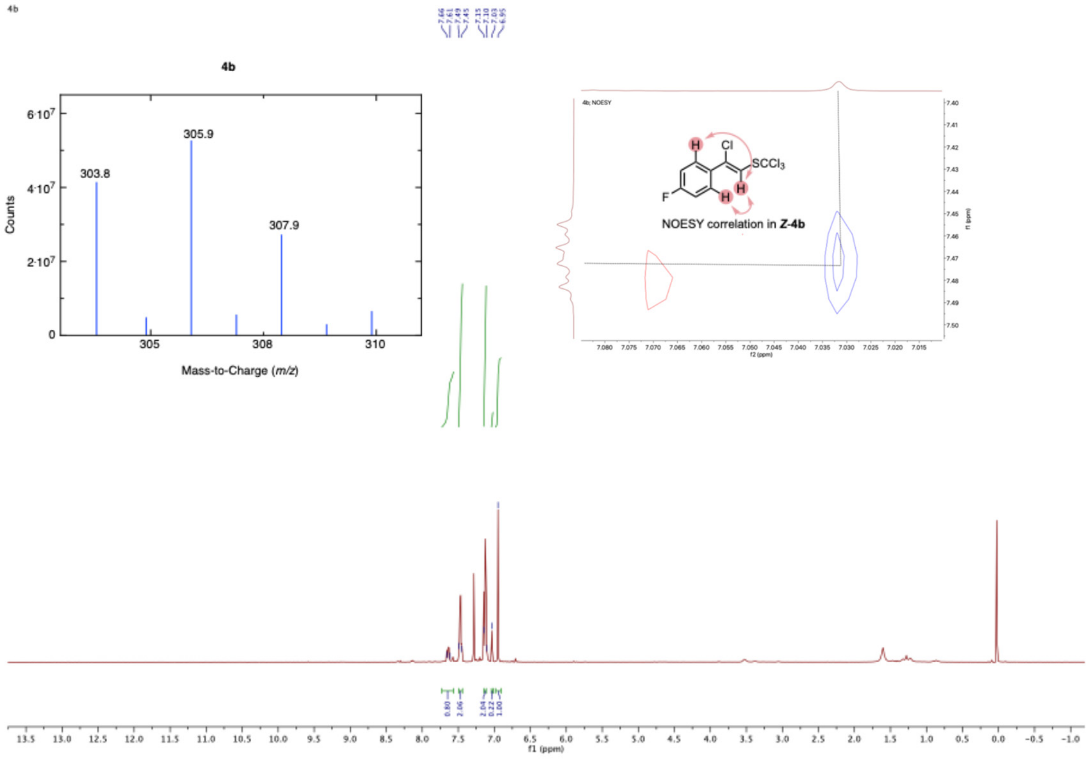
4b
Mass-to-Charge (m/z)
Counts
303.8
305.9
307.9
NOESY correlation in Z-4b
f1 (ppm)
f2 (ppm)

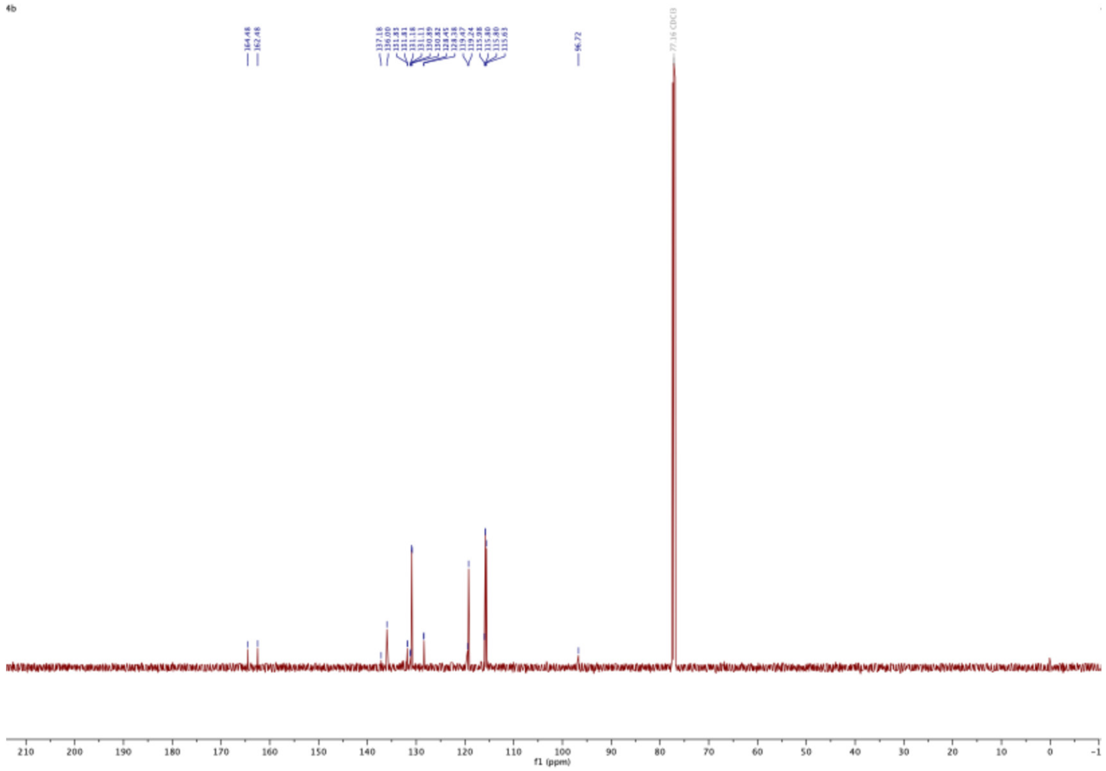
4b
f1 (ppm)

Determination of the light intensity at 450 nm:

The photon flux of a photoreactor (Penn PhD Photoreactor M2, Penn Photon Devices, LLC, Pennsburg, PA) equipped with a cooling system was measured using standard ferrioxalate actinometry following a procedure from the literature[1,2,3]. A 0.15 M solution of ferrioxalate was prepared by dissolving 2.21 g of potassium ferrioxalate hydrate in 30 mL of 0.05 M H_2SO_4. A buffered solution of phenanthroline was prepared by dissolving 50 mg of phenanthroline and 11.25 g of sodium acetate in 50 mL of 0.5 M H_2SO_4. Both solutions were stored in the dark. To determine the photon flux of the Penn PhD Photoreactor, 2.0 mL of the ferrioxalate solution was irradiated in the photoreactor for 30 seconds at a wavelength of 450 nm. After irradiation, 0.35 mL of the phenanthroline solution was added to the ferrioxalate solution. The solution was then allowed to rest in dark for 1 h to allow the ferrous ions to completely coordinate to the phenanthroline. The absorbance of the solution was measured at 510 nm. A non-irradiated sample was also prepared and the absorbance at 510 nm measured. Conversion was calculated using eq 1.

$$mol\ Fe^{2+} = \frac{V \bullet \Delta A}{l \bullet \varepsilon} \qquad (1)$$

Where V is the total volume (0.00235 L) of the solution after addition of phenanthroline, ΔA is the difference in absorbance at 510 nm between the irradiated and non-irradiated solutions, l is the path length (1.000 cm), and ε is the molar absorptivity at 510 nm (11100 L mol^{-1} cm^{-1}). The photon flux can be calculated using eq 2.

$$photon\ flux = \frac{mol\ Fe^{2+}}{\Phi \bullet t \bullet f} \qquad (2)$$

Where Φ is the quantum yield for the ferrioxalate actinometer (1.01 for a 0.15 M solution at λ = 436 nm), t is the time (30.0 s), and f is the fraction of light absorbed at λ = 450 nm (0.99833, *vide infra*). The photon flux was calculated (average of three experiments) to be 1,72 × 10^{-8} einstein s^{-1}.

Sample calculation:

$$mol\ Fe^{2+} = \frac{0{,}00235\ L \bullet 2{,}1223}{1{,}000\ cm \bullet 11100\ L\ mol^{-1} cm^{-1}} = 5{,}28\ x\ 10^{-7} mol$$

$$photon\ flux = \frac{5{,}28\ x\ 10^{-7} mol}{1{,}01 \bullet 30\ s \bullet 0{,}99833} = 1{,}745\ x\ 10^{-8}\ einstein\ s^{-1}$$

Determination of quantum yield:

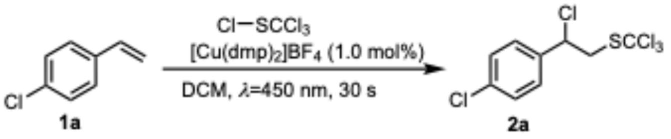

A glass vial was charged with $Cu(dmp)_2BF_4$ (0,005 mmol, 1.0 mol %) and 2 ml of DCM. The reaction mixture was then degassed using three freeze-pump-thaw (FPT) cycles. 4-chlorostyrene (0.5 mmol, 1 equiv) and trichloromethanesulfenyl chloride (0.75 mmol, 1,5 equiv) were then added under a nitrogen atmosphere. The sample was stirred and irradiated (λ = 450 nm) for 30 s. The yield of product formed was determined by ^{1}H NMR based on a 1,3,5-trimethoxybenzene as standard. The quantum yield was determined using eq 3. Essentially all incident light (f > 0.999, *vide infra*) is absorbed by the $Cu(dmp)_2BF_4$ at the reaction conditions described above.
Experiment 1: 2,9 mg (0,005 mmol) $Cu(dmp)_2BF_4$, 60 µL 4-chlorostyrene (0.5 mmol), 82 µL (0.75 mmol) trichloromethanesulfenyl chloride after 30 s yielded 7 % of product; Φ (7%) = 68

Sample quantum yield calculation:

$$\Phi = \frac{0{,}035\ x\ 10^{-3}\ mol}{1{,}72\ x\ 10^{-8} einstein\ s^{-1} \bullet\ 30\ s * 1{,}00} = 68 \qquad (3)$$

Reaction in the presence of TEMPO

A dried 8 mL vial was charged with **1a** (0.5 mmol), $[Cu(dmp)_2]BF_4$ (2.9 mg, 0,005 mmol, 1 mol %), TEMPO (78.0 mg, 0.5 mmol, 1 equiv and 165.3 mg, 1.0 mmol, 2 equiv), and CH_2Cl_2 (anhydrous, degassed, 2 mL) followed by addition of Cl_3CSCl (82 µL, 0.75 mmol). Vial was sealed off with a screw cap with a septum and solution degassed via tree freeze-pump-cycles. The resulting solution was irradiated with LED 450 nm for 2 h at 25 °C. The reaction mixture was quanched with Et_3N (105 µL, 0.75 mol) and solvent evaporated. The crude product was dissolved in $CDCl_3$ and analysed by ^{1}H NMR using CH_2Br_2 (28 µL, 0.4 mmol) as a standard. The NMR yield of **2a** was determined to be 54 % in the case of 1 equivalent of TEMPO and 23 % in the case of 2 equivalents of TEMPO. HRMS analysis revealed the presence of TEMPO adducts in both experiments.

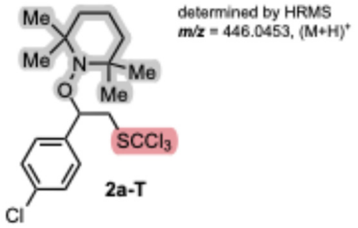

2a-T

DFT study

Density functional theory (DFT) calculations were performed using Gaussian 16 package, revision C.01.[4] All calculations were performed at the (U)ωB97X-D/Def2-TZVP/SMD(DCM) level of theory. Normal mode vibrational analisys on the stacionary points allowed us to confirme they are minima (zero imaginary frequencies) or transition state (TS, one imagynary frequncy). The Gibbs free energy values at 298.15 K and 1 atm were estimated by assuming ideal-gas, rigid-rotor, and harmonic-vibrational models. Intrinsic reaction coordinate (IRC) calculations were also carried out to verifie that they conect the expected minima. The 3D geometries in the manuscript were created with CYLview, 1.0b.[5]

To identify the potential transition state for radical $Cl_3C–S^{\bullet}$ addition to 4-chlorostirene **1a** elementary step, a relaxed scan of the potential energy surface along the reaction coordinate (S–C bond formation) at different angles of radical approach (Φ) was performed (Figure S1, b). The distance (*d*) was scanned in the range of 1.836 Å to 2.836 Å and an approach angle between 2° and 152° (Figure S1, b). The results indicate that Cl3C–S• radical addition is virtually barrier-free in nature (Figure S1, a).

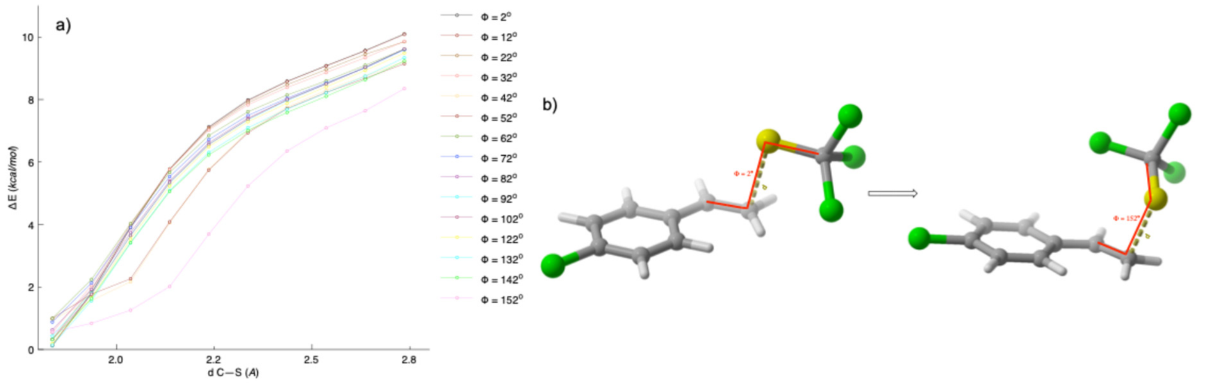

Figure S1. Figure S1. Relaxed scan of the potential energy surface along the reaction coordinate (S–C bond formation) to the intermediate **INT1**. Data were obtained at the (U)ωB97X-D/6-31+G(d,p)/SMD(DCM) level of theory.

Coordinate of the optimized structures

Cl_3CSCl

ωB97X-D/Def2-TZVP/SMD(DCM)

EE = –2277.130307

EE+ZPE = –2277.119019

Enthalpy = –2277.110810

Free Energy = –2277.152706

0 1

C	-0.52773	-0.03243	0.00002
S	0.82243	1.17214	0.00014
Cl	2.45006	-0.01777	-0.00002
Cl	-1.93955	1.04975	-0.00013
Cl	-0.54901	-1.06185	-1.44149
Cl	-0.54929	-1.06187	1.44150

1a

ωB97X-D/Def2-TZVP/SMD(DCM)

EE = –769.270106

EE+ZPE = –769.145199

Enthalpy = –769.136411

Free Energy = –769.178770

0 1

C	0.78193	1.24557	0.00000
C	-0.59990	1.35388	-0.00000
C	-1.41977	0.22600	-0.00001
C	-0.80919	-1.03010	-0.00001
C	0.56764	-1.15691	-0.00001
C	1.35311	-0.01394	-0.00000
H	1.40342	2.13110	0.00001
H	-1.05075	2.33944	0.00000
H	-1.41062	-1.93023	-0.00002

H	1.02737	-2.13624	-0.00001
C	-2.88052	0.40889	-0.00001
C	-3.80509	-0.54534	0.00002
H	-3.20576	1.44549	-0.00003
H	-4.85771	-0.29013	0.00002
H	-3.56107	-1.60176	0.00005
Cl	3.08975	-0.16976	0.00000

INT1

(U)ωB97X-D/Def2-TZVP/SMD(DCM)

EE = –2586.184271

EE+ZPE = –2586.048114

Enthalpy = –2586.032741

Free Energy = –2586.093724

0 2

C	-4.21670	1.06990	0.38276
C	-3.00935	1.72465	0.30186
C	-1.83607	1.06563	-0.14017
C	-1.95218	-0.30050	-0.49288
C	-3.15944	-0.95947	-0.41109
C	-4.28658	-0.27260	0.02456
H	-5.10267	1.59048	0.72143
H	-2.95073	2.76978	0.58113
H	-1.08403	-0.85321	-0.82820
H	-3.23100	-2.00460	-0.68188
Cl	-5.80937	-1.10570	0.12596

C	-0.62328	1.77961	-0.20998
C	0.67000	1.21056	-0.65864
H	-0.62079	2.81095	0.12120
H	0.54946	0.44343	-1.42314
H	1.32594	1.99480	-1.03290
S	1.47432	0.44539	0.80334
C	2.97653	-0.24469	0.09478
Cl	4.03560	0.99279	-0.64179
Cl	3.83851	-1.00654	1.45113
Cl	2.64980	-1.48740	-1.15049

TS1

(U)ωB97X-D/Def2-TZVP/SMD(DCM)

EE = –4863.301352

EE+ZPE = –4863.152547

Enthalpy = –4863.128502

Free Energy = –4863.213234

ν_{imag} = 1 (–379.9 cm^{-1})

0 2

C	-3.18283	-2.84022	-1.20044
C	-2.27148	-1.80909	-1.29370
C	-1.67146	-1.26610	-0.14854
C	-2.00772	-1.80232	1.10248
C	-2.91697	-2.83428	1.20690
C	-3.49800	-3.34440	0.05349
H	-3.64552	-3.24896	-2.08838

H	-2.01762	-1.40775	-2.26734
H	-1.55329	-1.41932	2.00681
H	-3.17414	-3.24307	2.17453
Cl	-4.64333	-4.64240	0.18450
C	-0.72318	-0.18901	-0.30651
C	-0.37910	0.75703	0.79780
H	-0.66639	0.22941	-1.30355
H	-0.12321	0.23610	1.72207
H	0.47146	1.38088	0.53153
S	-1.74804	1.83189	1.33394
C	-1.93587	3.02812	-0.00334
Cl	-2.67092	2.34166	-1.48304
Cl	-3.04807	4.25333	0.65116
Cl	-0.40539	3.81304	-0.46489
Cl	1.24876	-1.24517	-0.51565
S	3.40061	-1.95978	-0.63459
C	4.21734	-0.51978	0.04759
Cl	5.95271	-0.94030	0.01386
Cl	3.96647	0.96381	-0.91317
Cl	3.74583	-0.16519	1.73314

2a

ωB97X-D/Def2-TZVP/SMD(DCM)

EE = –3046.444145

EE+ZPE = –3046.303574

Enthalpy = –3046.286953

Free Energy = –3046.351673

0 1

C	3.44751	-0.28040	-1.21480
C	2.41497	0.63375	-1.36136
C	1.59425	0.95995	-0.29012
C	1.82354	0.36117	0.94512
C	2.84948	-0.55132	1.10839
C	3.65214	-0.86422	0.02171
H	4.08140	-0.53224	-2.05423
H	2.24870	1.09439	-2.32799
H	1.20329	0.60803	1.79834
H	3.02376	-1.01526	2.06988
Cl	4.94453	-2.01518	0.22133
C	0.47109	1.93917	-0.49221
C	-0.89941	1.45937	-0.02726
H	0.42948	2.23776	-1.53771
H	-0.86190	1.15332	1.01783
H	-1.63092	2.25604	-0.14489
S	-1.39866	0.03840	-1.04471
C	-2.68802	-0.68375	-0.02163
Cl	0.81902	3.48631	0.39797
Cl	-4.02639	0.44890	0.31796
Cl	-3.31295	-2.05685	-0.96071
Cl	-2.07764	-1.29017	1.54622

Literature

[1] Hatchard, C. G.; Parker, C. A. Proc. Roy. Soc. (London) 1956, A235, 518–536.

[2] Kuhn, H. J.; Braslavsky, S. E.; Schmidt, R. Pure Appl. Chem. 2004, 76, 2105–2146.
[3] Monalti, M. et. al. Chemical Actinometry. Handbook of Photochemistry, 3rd Ed; Taylor & Francis Group, LLC. Boca Raton, FL, 2006, 601–616.
[4] (a) Y. Zhao, D. G. Truhlar, *Theor. Chem. Acc.* **2008**, *120*, 215–241; (b) P. C. Hariharan, J. A. Pople, *Theor. Chim. Acta* **1973**, *213*, 213–222. (c) Gaussian 16, Revision C.01, M. J. Frisch, G. W. Trucks, H. B. Schlegel, G. E. Scuseria, M. A. Robb, J. R. Cheeseman, G. Scalmani, V. Barone, B. Mennucci, G. A. Petersson, H. Nakatsuji, M. Caricato, X. Li, H. P. Hratchian, A. F. Izmaylov, J. Bloino, G. Zheng, J. L. Sonnenberg, M. Hada, M. Ehara, K. Toyota, R. Fukuda, J. Hasegawa, M. Ishida, T. Nakajima, Y. Honda, O. Kitao, H. Nakai, T. Vreven, J. A. Montgomery, Jr., J. E. Peralta, F. Ogliaro, M. Bearpark, J. J. Heyd, E. Brothers, K. N. Kudin, V. N. Staroverov, R. Kobayashi, J. Normand, K. Raghavachari, A. Rendell, J. C. Burant, S. S. Iyengar, J. Tomasi, M. Cossi, N. Rega, J. M. Millam, M. Klene, J. E. Knox, J. B. Cross, V. Bakken, C. Adamo, J. Jaramillo, R. Gomperts, R. E. Stratmann, O. Yazyev, A. J. Austin, R. Cammi, C. Pomelli, J. W. Ochterski, R. L. Martin, K. Morokuma, V. G. Zakrzewski, G. A. Voth, P. Salvador, J. J. Dannenberg, S. Dapprich, A. D. Daniels, O. Farkas, J. B. Foresman, J. V. Ortiz, J. Cioslowski, D. J. Fox, Gaussian Inc., Wallingford CT, **2009**.
[5] CYLview20, C. Y. Legault, Université de Sherbrooke, 2020 (http://www. cylview.org).
